# GLP‐1 receptor agonists and the risk for cancer: A meta‐analysis of randomized controlled trials

**DOI:** 10.1111/dom.16489

**Published:** 2025-05-29

**Authors:** Giovanni Antonio Silverii, Christian Marinelli, Costanza Bettarini, Gloria Giovanna Del Vescovo, Matteo Monami, Edoardo Mannucci

**Affiliations:** ^1^ AOU Careggi, Diabetology Unit – Experimental and Clinical Biomedical Sciences “Mario Serio” Department University of Florence Florence Italy; ^2^ Diabetology Unit AOU Careggi Florence Italy

**Keywords:** cancer, dulaglutide, exenatide, GLP‐1 RA, glucagon‐like peptide‐1 receptor agonists, liraglutide, meta‐analysis, obesity, semaglutide, type 2 diabetes mellitus

## Abstract

**Aims:**

To assess if there is a difference in the oncogenic risk between GLP‐1 RA and comparators in randomized controlled trials.

**Materials and Methods:**

A meta‐analysis of randomized controlled trials comparing GLP‐1RA to any comparators for diabetes and/or obesity, lasting at least 52 weeks. The endpoints included the incidence of overall cancers and single malignancies.

**Results:**

Fifty trials were included. GLP‐1RA treatment was not associated with a significant difference in risk for overall cancer (MH‐OR 1.05, 95% confidence interval [CI] [0.98, 1.13]). Uterine cancer was significantly reduced in the GLP‐1RA arm in trials performed in subjects with obesity (MH‐OR 0.24, 95% CI [0.06, 0.94]), but not in those aimed at diabetes treatment (MH‐OR 0.92, [0.58, 1.47]). We detected an increase in the risk for thyroid cancer (MH‐OR 1.55, [1.05, 2.27]), more evident in longer‐term trials, and in the risk for colorectal cancer (MH‐OR 1.27 [1.03, 1.57]), which, conversely, was significant only in shorter‐term trials. No significant difference in the risk was detected for any other cancer.

**Conclusions:**

GLP‐1 RA do not appear to produce an effect on most malignancies in clinical trials. A reduction of very close obesity‐associated cancers seems possible, whereas a risk signal for thyroid cancer was observed, prompting the need for further specific studies. On the other hand, the small increase observed in colorectal cancer in shorter‐term trials may be the effect of a disproportionate increase in diagnostic procedures in the GLP‐1 RA arm, because of the suspicion raised by common side effects of GLP‐1 RA.

## 1 INTRODUCTION

Type 2 Diabetes mellitus (T2DM) and obesity have both been associated with an increased incidence of cancer.^1^, ^2^, ^3^ The risk is further increased in individuals with both T2DM and obesity^4^ and in long‐term obesity.^5^ In particular, 13 malignancies have been identified as obesity‐associated cancers, for which the evidence is sufficient to identify obesity as a risk factor^6^; overall, weight excess may cause up to 20% of total cancer mortality.^7^

The possible pathogenetic mechanisms implied seem to be different for each neoplasm^8^: both altered adipocyte function and insulin resistance determine an increase in circulating levels of hormones, such as oestrogens, insulin and insulin‐like growth factors, which may in turn enhance tumour proliferation and inhibit apoptosis^8^; furthermore, adipokines may contribute to oncogenesis by stimulating chronic inflammatory mediators, increasing oxidative stress and impairing immune responses.^9^ Excess weight is also associated with conditions, such as gastroesophageal reflux and steatotic liver disease, which in turn increase the risk of specific cancers (oesophageal adenocarcinoma and hepatocellular carcinoma, respectively). Conversely, intentional weight loss has been associated with a reduction in oncogenic risk.^10^ Long‐term observations that patients undergoing bariatric surgery show a reduced incidence of cancer in comparison with age‐ and sex‐matched subjects^11^ suggest that treatment of obesity, when producing a relevant weight loss, could reduce the incidence of malignancies.

The relationship between diabetes and cancer is similarly complex. The association between diabetes and incident malignancies has been extensively reported for type 2 diabetes, whereas data on type 1 diabetes are less impressive.^12^ Some associations (e.g., type 2 diabetes and pancreatic cancer) could be partly due to reverse causation, with the still undiagnosed incident malignancy inducing hyperglycaemia.^13^ Furthermore, the effect of confounders (particularly, visceral adiposity) could play a major role in the epidemiologic association of type 2 diabetes with cancer. It is also possible that hyperinsulinaemia contributes to the risk of malignancies in people with diabetes.^14^, ^15^ There is no clear evidence of an association between higher A1c levels in people with diabetes and a greater risk of cancer.^16^ In clinical trials, intensified treatment of type 2 diabetes, leading to improved glycaemic control, did not reduce cancer incidence,^16^ but the sample size and duration of available trials could have been insufficient for this endpoint.

Glucagon‐like peptide‐1 receptor agonists (GLP‐1 RA) are very effective in improving glucose control in people with diabetes and achieving weight loss in subjects with overweight or obesity,^17^, ^18^ while also reducing cardiovascular adverse events in these high‐risk populations.^19^, ^20^ It is plausible that GLP‐1 RA also reduce the incidence of obesity‐associated cancers, as recently suggested by an observational study^21^; on the other hand, another investigation failed to detect any effect of these drugs on the incidence of cancer.^22^

Conversely, the possible detrimental effects of GLP‐1RA on the risk of some specific malignancies are still controversial. The Food and Drug Administration and the European Medicines Agency state that GLP‐1RA should be used with caution in patients with a personal or familial history of medullary thyroid cancer.^23^, ^24^ This statement is based only on preclinical (rodent) data, without further clinical evidence.^25^, ^26^ Furthermore, a possible increase in overall thyroid cancer in GLP‐1RA users has been reported in epidemiological studies^27^, ^28^ and clinical trials,^29^ whereas other observational studies failed to detect either beneficial or detrimental effects of GLP‐1RA on this endpoint.^21^, ^30^, ^31^

The results of observational studies, although interesting, can never be conclusive, because of the inevitable effect of confounders. Although some of the studies^21^, ^27^ provided adjusted analyses incorporating the effects of many concurrent parameters, the possibility of residual confounding can never be excluded. Therefore, results of observational studies should be verified through the analysis of data from randomized trials.

Data from individual trials do not show significant between‐group differences for any malignancy, but the number of events recorded in each study is too small to draw any conclusion. A previous meta‐analysis^32^ did not detect any significant association between GLP1‐RA and cancer incidence, but recent long‐term trials,^33^, ^34^ which were not available at the time, may have added new information on this topic.

The assessment of the effects of GLP‐1RA on cancer incidence is relevant for clinical practice. The demonstration of favourable effects, if any, would strengthen the motivation to choose these drugs, instead of possible therapeutic alternatives, in the treatment of obesity and type 2 diabetes. Conversely, an increased incidence of some types of cancer, if present, should be considered in the assessment of the risk–benefit ratio, particularly when treating relatively healthy overweight/obese patients.

The present meta‐analysis aims to summarize all the evidence from randomized trials on the effects of GLP‐1RA on the incidence of different malignancies.

## 2 MATERIALS AND METHODS

This is a post hoc analysis of a review aimed at assessing the incidence of thyroid malignancies, previously registered on the PROSPERO website (registration number CRD42023456382).^35^ The present meta‐analysis was conducted following the Preferred Reporting Items for Systematic Reviews and Meta‐Analyses reporting guidance.^36^

### 2.1 Data sources and search strategy

A systematic search was performed, which encompassed the PubMed, Embase, Clinicaltrials.gov and Cochrane CENTRAL Database databases, up to November 25th, 2024, including all the GLP‐1RA drug names as keywords, including only reports on studies on humans, which were written in English, French, Spanish or Italian language. We reported the full search strings in Table S1.

### 2.2 Endpoints

The principal endpoint was the incidence of any malignant neoplasia during the study, reported as a serious adverse event, and the incidence of each of the tumours known as associated with obesity (*Uterine cancer, Oesophageal cancer, Thyroid cancer, Gastric cancer, Colorectal cancer, Liver cancer, Gallbladder cancer and Cholangiocarcinoma, Pancreatic cancer, Breast cancer, Ovary cancer, Kidney cancer, Meningioma, Multiple myeloma*)^6^; secondary endpoints were the incidence of each other malignancy, reported as serious adverse events.

### 2.3 Study selection

We included only randomized controlled trials designed to compare any of the GLP‐1RA currently approved by European Medical Agency, for any approved indication (i.e.T2DM or obesity), with placebo or any comparator, with the exception of other GLP‐1RA and GLP‐1/GIP and GLP‐1/Glucagon dual agonists, regardless of the study primary endpoint, provided that at least a case of malignancy was detected and reported in the study. Studies lasting less than 52 weeks were excluded, as well as studies performed in patients younger than 18 years and studies that did not report a full list of adverse events.

### 2.4 Data extraction

The variables of interest were the incidence of any malignancy at endpoint, trial duration, age at baseline, body mass index (BMI) at baseline and percentage of females enrolled. If available, the estimates for each variable were extracted from the principal publication in a pre‐determined electronic sheet. Whenever needed, secondary publications and clinicaltrials.gov registry were used for retrieval of missing information, in the hierarchical order reported above. Four authors (G.A.S., C.M., C.B., G.G.D.V.) independently extracted data, whereas conflicts were resolved by a fifth investigator (M.M.).

### 2.5 Data analysis and quality assessment

Two of the investigators (G.A.S. and C.M.) assessed the risk of bias in RCTs using the revised Cochrane recommended tool,^37^ which includes five specific domains: (1) bias arising from the randomization process; (2) bias due to deviations from intended interventions; (3) bias due to missing outcome data; (4) bias in the measurement of the outcome; (5) bias in the selection of the reported result.

The results of these domains were graded as ‘low’ risk of bias, ‘high’ risk of bias or ‘uncertain’ risk of bias. The aim was to assess the effect of assignment to intervention (the ‘intention‐to‐treat’ effect). For domains 3, 4, 5, the present meta‐analysis's primary endpoint, that is, the incidence of malignancies, was considered as the outcome of interest. For each domain all the ‘signalling questions’ comprised in the tool were addressed, a judgement about risk of bias for the domain was made, as well as an overall risk of bias judgement for the study. Conflicts between the judgements of the two investigators were resolved by a third investigator (MM).

Mantel‐Haenzel Odds Ratio (MH‐OR) for categorical variables was calculated using random effect models in case of significant heterogeneity, whereas a fixed‐effects model was used if heterogeneity was not relevant; a sensitivity analysis was performed using a fixed‐effects model in the case of significant heterogeneity, or vice versa. In addition, a further sensitivity analysis was performed after excluding open‐label trials. All these analyses excluded studies with zero events.

The statistical power was calculated, post hoc, for overall cancer incidence in the control group, using the method proposed by Schoenfeld.^38^ Statistical heterogeneity was assessed by *I* ^2^ test, whereas funnel plots were used to detect publication bias for principal endpoints with at least 10 trials. Subgroup analyses were performed for trials using different molecules, in the hypothesis that effects on cancer could be drug‐specific, rather than common to the whole class. A further subgroup analysis was performed on the basis of trial duration (52, 53–102 and >102 weeks); if exposure to treatment is associated with a change in the incidence of malignancies, such an effect should be expected to be more evident in longer‐term studies. In addition, a subgroup analysis was performed for trials designed for the treatment of either obesity or type 2 diabetes; for obesity, GLP‐1RA doses are higher than for diabetes, and the case mix is quite different, with possible differences of effects on cancer. The results of individual studies and the syntheses of meta‐analyses will be displayed as forest plots. All analyses were performed using Review Manager (RevMan), Version 5.4.1 (Copenhagen: The Nordic Cochrane Centre, The Cochrane Collaboration).

## 3 RESULTS

### 3.1 Characteristics of included trials

We retrieved 6036 items after removing duplicates. Among those, we selected 99 records for full text retrieval, of which 50 studies fulfilled all the inclusion criteria, overall including 55,305 and 47,467 patients in GLP‐1RA and placebo arms, respectively. Of those 50 trials, 37 included only patients with type 2 diabetes, whereas 13 were performed enrolling obese non‐diabetic individuals; liraglutide, semaglutide, exenatide, dulaglutide and lixisenatide were used in 19, 9, 5 and 1 trials, respectively. The graphical trial research flow summary was displayed in Figure S1; we reported the characteristics of the included trials in Table 1, and the excluded studies in Table S2. The median duration of the studies was 65 weeks, whereas the mean duration of treatment in enrolled patients was 141.2 weeks. The median age was 57 years, and the median BMI was 32 kg/m^2^. The Risk of bias table and summary are reported in Figures S2 and S3, respectively. All studies except for seventeen^39^, ^40^, ^41^, ^42^, ^43^, ^44^, ^45^, ^46^, ^47^, ^48^, ^49^, ^50^, ^51^, ^52^, ^53^, ^54^, ^55^ were double‐blind.

**TABLE 1:** Characteristics of the included trials.

Study	Ndrug	Ncomp	Indication	Drug	Comparator	Dur	Age	BMI
Ahren^69^	818	407	DM	Semaglutide	Sitagliptin	56	55.0	32.5
Aroda^39^	506	506	DM	IdegLira	Glargine	104	56.6	32.0
Astrup^40^	369	193	OB	Liraglutide	Orlistat	104	45.5	34.8
Blonde^41^	588	296	DM	Dulaglutide	Glargine	52	59.5	32.5
Buse^42^	253	98	DM	Semaglutide	Sita	52	57.0	31.0
Davies^76^	211	212	DM	Liraglutide	Placebo	56	55.0	37.4
Davies^77^	807	403	DM	Semaglutide	Placebo	68	55.0	35.7
Gallwitz^43^	490	487	DM	Exenatide	Glimepiride	208	56.0	32.4
Garber^78^	498	248	DM	Liraglutide	Glimepiride	104	53.0	33.0
Garvey^79^	195	197	DM	Liraglutide	Placebo	56	Nr	Nr
Garvey^80^	152	152	OB	Semaglutide	Placebo	104	47.0	38.5
Gerstein^57^	4949	4952	DM	Dulaglutide	Placebo	281	66.0	32.3
Giorgino^45^	545	262	DM	Dulaglutide	Glargine	78	57.0	31.3
Gough^44^	414	413	DM	Liraglutide	Degludec	52	55.0	31.2
Holman^81^	7356	7396	DM	Exenatide	Placebo	166	62.0	31.7
Husain^82^	1591	1592	DM	Semaglutide	Placebo	68	66.0	32.0
Jaiswal^46^	22	24	DM	Exenatide	Glargine	78	52.0	36.0
Kadowaki^83^	401	101	OB	Semaglutide	Placebo	68	50.0	nr
Kaku^47^	480	121	DM	Semaglutide	NONE	56	58.0	27.4
Knop^84^	334	333	OB	Semaglutide	Placebo	75	50.0	37.5
Kosiborod^85^	263	266	DM	Semaglutide	Placebo	52	69	37
Lincoff^33^	8803	8801	OB	Semaglutide	Placebo	170	62	33
Marso^86^	1648	1649	DM	Semaglutide	Placebo	109	65.0	32.8
Marso^56^	4668	4672	DM	Liraglutide	Placebo	198	64.0	32.5
Miyagawa^87^	281	70	DM	Dulaglutide	Placebo	52	58.0	25.0
Nahra^48^	110	112	OB	Liraglutide	Placebo	54	56.0	34.8
Nauck^49^	253	248	DM	Exenatide	Insulin	52	58.0	30.4
Nauck^88^	483	363	DM	Liraglutide	Glimepiride	104	57.0	31.2
O'Neill^89^	103	136	OB	Liraglutide	Placebo	52	47	39.3
Perkovic^34^	1767	1766	DM	Semaglutide	Placebo	177	32	32
Pfeffer^90^	3034	3034	DM	Lixisenatide	Placebo	107	60.0	30.1
Pi‐sunyer^91^	2481	1242	OB	Liraglutide	Placebo	70	45.0	38.3
Pratley^50^	439	219	DM	Liraglutide	Sitagliptin	52	55.0	32.8
Pratley^92^	284	142	DM	Liraglutide	Placebo	52	56.0	33.0
Rodbard^51^	410	409	DM	Semaglutide	Empagliflozin	52	58.0	32.8
Rosenstok^93^	1397	467	DM	Semaglutide	Placebo	78	58.0	32.5
Rubino^52^	253	85	OB	Liraglutide	Placebo	52	49.0	37.5
Ruff^94^	2074	2070	DM	Exenatide	Placebo	62	63.0	32.3
Tuttle^53^	383	194	DM	Dulaglutide	Glargine	52	64.5	32.3
Umpierrez^95^	269	268	DM	Dulaglutide	Metformin	52	56.0	33.3
Unger^54^	980	984	DM	Liraglutide	Any	104	57.4	33.5
Wadden^96^	212	210	OB	Liraglutide	Placebo	56	46.2	37.9
Wadden^97^	142	140	OB	Liraglutide	Placebo	56	47.2	39.0
Wadden^98^	407	204	OB	Semaglutide	Placebo	68	46.0	38.0
Wang^55^	515	259	DM	Dulaglutide	Glargine	52	55.0	26.0
Wang^99^	16	9	DM	Dulaglutide	Glargine	52	62.0	25.0
Weinstock^100^	606	177	DM	Dulaglutide	Placebo	104	54.0	31.0
Wilding^101^	1306	655	OB	Semaglutide	Placebo	68	46.0	37.9
Yamada^102^	195	49	DM	Semaglutide	Placebo	52	61.0	26.0
Zinman^103^	362	184	DM	Semaglutide	Placebo	52	61.0	31.0

Abbreviations: Age, mean age of participants (years); BMI, mean body mass index of enrolled patients (in kg/m^2^); DM, diabetes mellitus; Dur, duration of the study (weeks); Ind, indication; OB, obesity.

In summary, all the trials showed the possibility of missing outcome data, because the incidence of cancers was not a pre‐specified endpoint; 15 trials harboured possible issues related to the measurement of the outcome, 14 trials reported a bias related to the measurement of the outcome and 12 trials showed possible bias in reported results, whereas only one trial did not fully rule out concerns related to the randomization process.

### 3.2 Overall cancer

**FIGURE 1:**
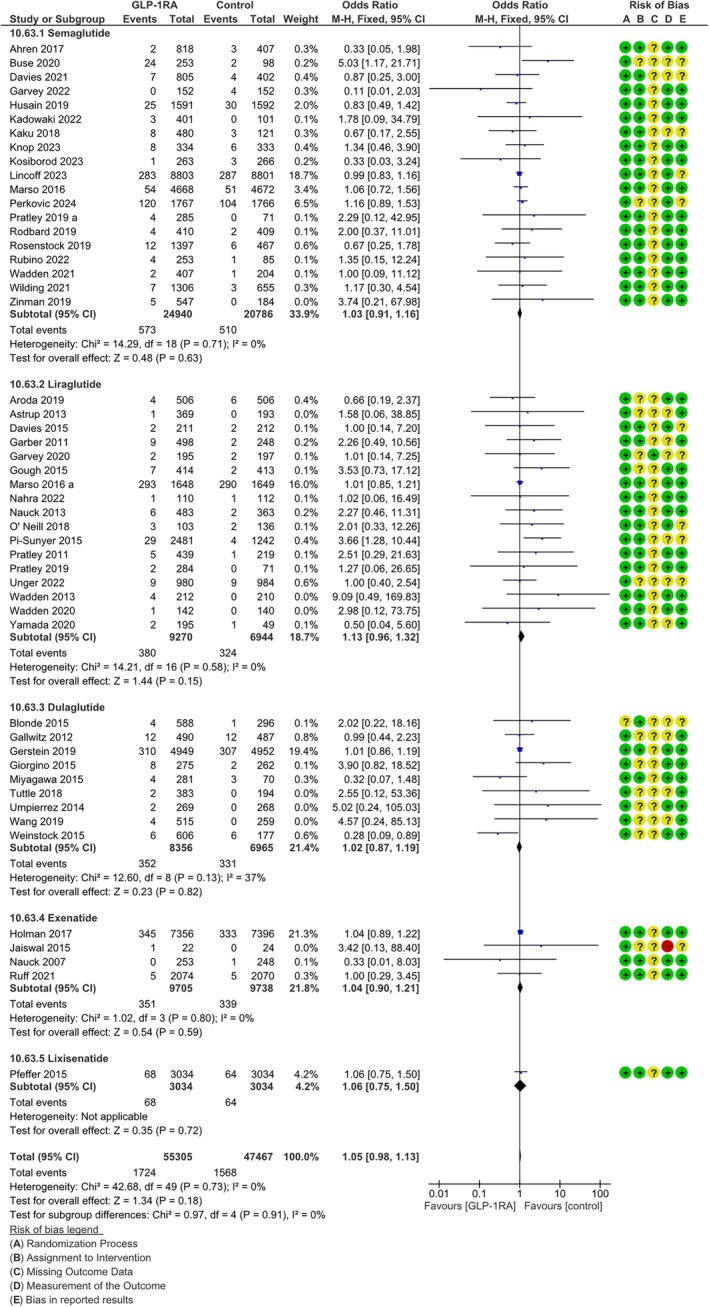
Difference in risk for any cancer between patients on GLP‐1RA and patients on comparators (forest plot). CI, confidence interval; M‐H, Mantel‐Haenzel.

**FIGURE 2:**
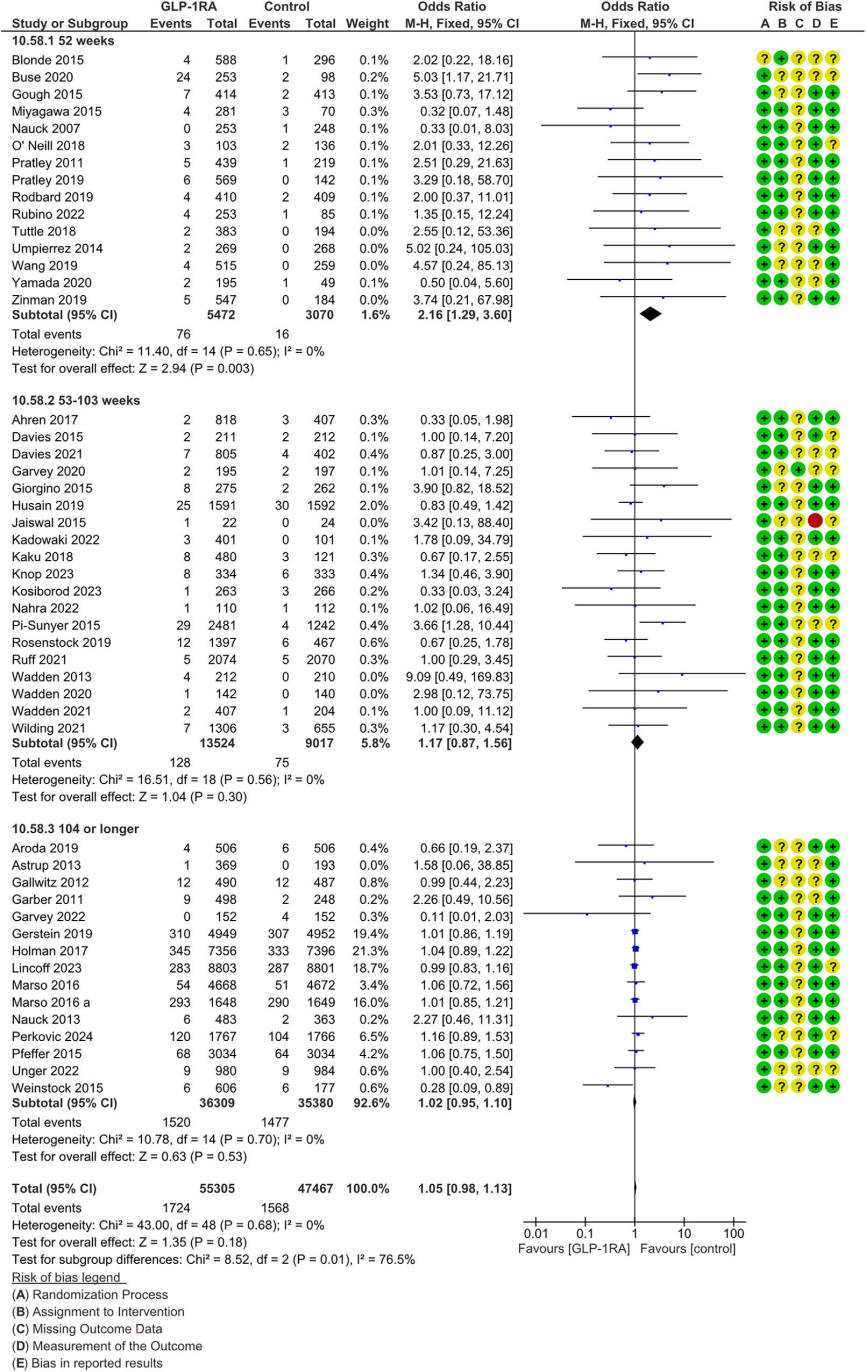
Difference in risk for any cancer between patients on GLP‐1RA and patients on comparators (forest plot); subgroup analysis between trials lasting 52 weeks, trials lasting more than 53 but less than 103 weeks, and trials lasting 104 weeks or more. CI, confidence interval; M‐H, Mantel‐Haenzel.

We detected 1724 malignancies in the GLP‐1RA arm and 1568 in the comparator arm. With this number of events, the probability of detecting as statistically significant a 10% difference in the overall incidence of malignancies between the two arms would be 15%. The visual analysis of the funnel plot for overall cancer did not suggest any risk for publication bias (Figure S4). GLP‐1RA treatment was not associated with a significant difference in risk for overall cancer in the fixed‐effects analysis (MH‐OR 1.05, 95% CI [0.98, 1.13], *p* = 0.69, Figure 1), without any heterogeneity (*I* ^2^ = 0%). Sensitivity analyses performed with a random effects model (MH‐OR 1.04, 95% CI [0.97, 1.11]) and excluding open‐label trials (MH‐OR 1.03, 95% CI [0.95, 1.10]) provided similar results. In subgroup analyses, no difference in effect was detected between trials performed with different molecules of the class, or between studies performed on subjects with a mean baseline BMI higher or lower than 35 kg/m^2^ (*p* = 0.91 and *p* = 0.16 for difference between different molecules, respectively, Figures 1 and S6). When analysing trials with different durations separately, a statistically significant difference across groups was detected (*p* = 0.01, Figure 2): a significant positive association of GLP‐1RA with the incidence of cancer was found in the trials lasting 52 weeks (MH‐OR 2.20, 95% CI [1.32, 3.64], *p* = 0.003, Figure 2), whereas the difference was not significant for trials with a duration between 53 and 103 weeks (MH‐OR 1.17, 95% CI [0.87, 1.56], Figure 2), and no difference was found between groups for trials lasting at least 104 weeks (MH‐OR 1.02, 95% CI [0.95, 1.10], Figure 2).

### 3.3 Incidence of obesity‐associated cancers

#### 3.3.1 Uterine cancer

**FIGURE 3:**
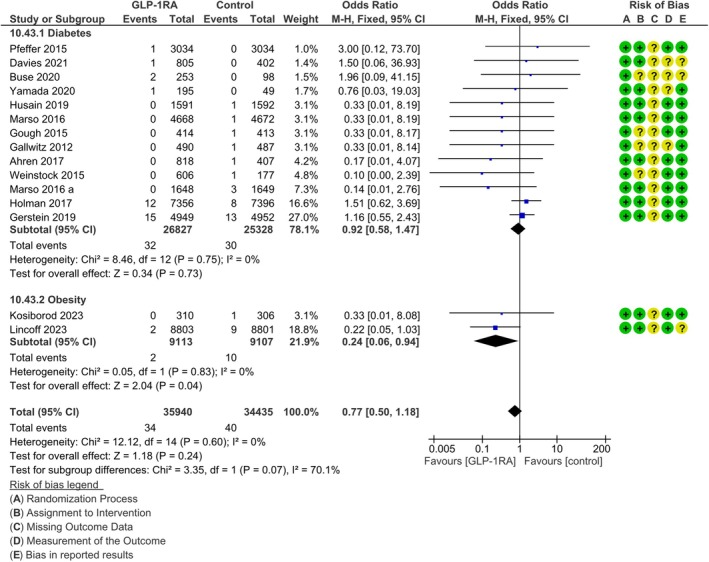
Difference in risk for endometrial cancer between patients on GLP‐1RA and patients on comparators (forest plot); subgroup analysis between trials. CI, confidence interval; M‐H, Mantel‐Haenzel.

We retrieved 74 cases of uterine cancer in 15 studies. No significant publication bias was suggested by the Funnel plot (Figure S5). The difference between GLP‐1RA and the comparator did not reach statistical significance (MH‐OR 0.77, 95% CI [0.50, 1.18], *p* = 0.24, *I* ^2^ = 0%, Figure 3). However, a subgroup analysis showed that, in trials performed with subjects with obesity, there was a significant reduction of uterine cancers in the GLP‐1RA arm (MH‐OR 0.24, 95% CI [0.06, 0.94], Figure 3), whereas no significant difference was found in trials performed in those with diabetes (MH‐OR 0.92, 95% CI [0.58, 1.47], Figure 3), although the difference between the two groups did not reach statistical significance (*p* = 0.07).

#### 3.3.2 Oesophageal cancer

**FIGURE 4:**
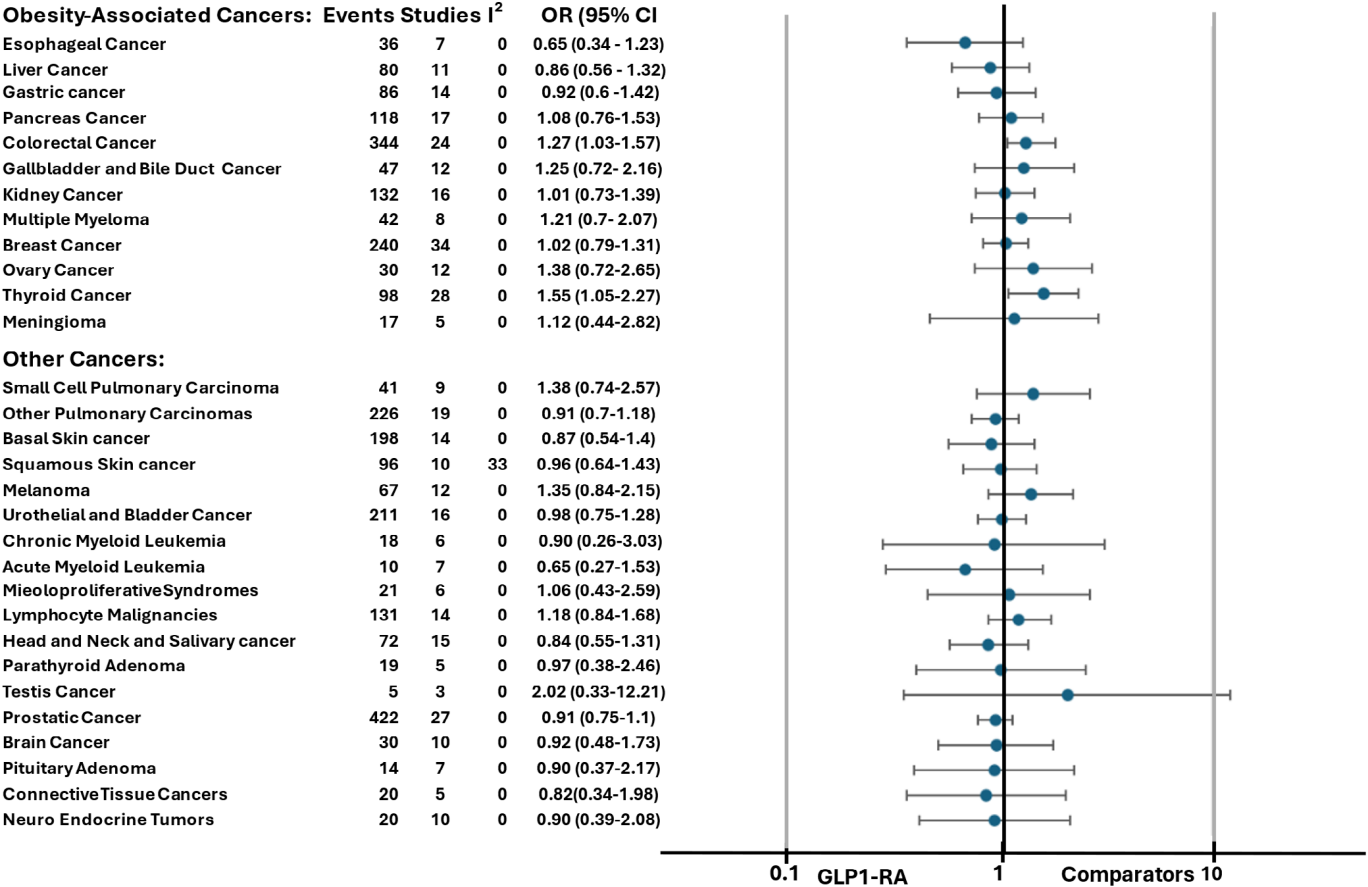
Difference in risk for each other specific cancer between patients on GLP‐1RA and patients on comparators (forest plot); CI, confidence interval; OR, odds ratio; please note that “Lymphocyte Malignancies” encompasses both Lymphomas and Chronic Lymphatic Leukaemia.

Seven trials reported at least one case of oesophageal malignant neoplasm; differences between GLP‐1RA and comparator arms did not reach statistical significance (MH‐OR 0.65, 95% CI [0.34, 1.23], *p* = 0.19, *I* ^2^ = 0%, Figures 4 and S7).

#### 3.3.3 Thyroid cancer

We retrieved 94 cases of thyroid cancer in 28 studies enrolling 46 412 and 41 053 patients in GLP‐1RA and comparator groups, respectively, with a mean duration of treatment of 90.3 weeks (125 235 and 117 303 patient*years in treatment and comparator groups, respectively), without any suspected publication bias (Figure S8). In another 22 studies, enrolling 10 336 and 6904 patients in GLP‐1RA and comparator groups, respectively, with a mean duration of treatment of 77 weeks (18351and 14 023 patient*years in treatment and comparator groups, respectively), no cases of thyroid cancer were reported. A significant increase in the incidence of thyroid cancers was observed in GLP‐1 RA arms (MH‐OR 1.55, 95% CI [1.05, 2.27], *p* = 0.03, *I* ^2^ = 0%, Figures 4 and S9), without any significant difference between different drugs (*p* = 0.79 for subgroup differences, Figure S10). We performed a sensitivity analysis excluding open‐label trials, obtaining similar results (MH‐OR 1.52, 95% CI [1.02, 2.27], *p* = 0.04, *I* ^2^ = 0). Notably, the effect of GLP‐1RA on thyroid cancer was significant only in trials with longer durations. However, the difference across groups of trials with different durations did not reach statistical significance (Figure S9).

#### 3.3.4 Colorectal cancer

Fifteen studies reported at least one case of colorectal cancer, without any sign of publication bias (Figure S11). The incidence of colorectal cancer was higher in the GLP‐1RA arm (MH‐OR 1.27 [1.03, 1.57], *p* = 0.03, *I* ^2^ = 0%, Figures 4 and S12); notably, the difference between treatment arms was statistically significant in shorter (up to 104 weeks) trials (MH‐OR 2.28 [1.04, 4.98]; *p* = 0.04), but not in those longer than 104 weeks (MH‐OR 1.20 [0.96, 1.50]), although the difference between groups of trials did not reach statistical significance (*p* = 0.12, Figure S12); the difference between different molecules was not significant as well (*p* = 0.98, Figure S13).

#### 3.3.5 Other obesity‐associated malignancies

Results obtained for the other obesity‐associated malignancies (i.e., gastric cancer, gallbladder and bile duct cancer, liver cancer, pancreatic cancer, breast cancer, ovary cancer, kidney cancer, meningioma, multiple myeloma) are summarized in Figure 4 and reported in detail in Figures S15, S17, S19, S21, S23, S25, S27–S29. No significant association of GLP‐1RA treatment was detected for any of those neoplasms, with low heterogeneity between studies. Funnel plots performed for outcomes retrieved in at least 10 studies ruled out the possibility of publication bias (Figures S14, S16, S18, S20, S22, S24, S26).

### 3.4 Cancers without established link to obesity

No difference between GLP‐1RA and comparator arms was detected in the incidence of other types of cancer (prostate cancer; testis cancer; urothelial and bladder cancer; basal, and squamous skin cancers; melanomas; non‐small cells, and small cells, pulmonary cancers; head and neck and salivary cancer; acute, and chronic myeloid leukaemias; lymphatic malignancies, parathyroid adenomas; brain cancers; connective tissue cancers; neuroendocrine tumours), as reported in Figure 4 in summary and in Figures S30–S42 in detail. The heterogeneity between studies was low for all the outcomes.

## 4 DISCUSSION

Randomized trials have already shown that GLP‐1RA reduce cardiovascular morbidity and mortality in people with obesity and/or diabetes.^33^, ^56^, ^57^ However, they still fail to demonstrate a major beneficial effect on incident cancer, another long‐term outcome related to obesity and diabetes.

Indeed, among the obesity‐related cancers, only a reduction in the incidence of uterine cancer in trials performed in obese non‐diabetic subjects was detected, whereas the effect was not significant for studies in diabetes. It is possible that beneficial effects on cancer risk are more evident with higher doses of those molecules that produce a greater weight loss, which are indicated for the treatment of obesity, although other mechanisms cannot be ruled out. However, the difference between trials on obesity and diabetes was not statistically significant; the limited number of observed cases prevents a more accurate assessment of the effects of treatment in different patient populations. In fact, trials on obesity were performed on subjects with a higher baseline body mass index, using drugs (i.e., liraglutide or semaglutide) that produce a greater weight loss than other GLP‐1RA,^58^ and with higher doses than those used for diabetes. On the other hand, the result observed in trials on obesity should be interpreted cautiously because of the limited number of observed events, which were largely retrieved from one single trial.^33^ In addition, this result was observed in a post hoc analysis, which should be considered hypothesis‐generating, needing to be further confirmed.

Among different neoplasms, those with a greater association with obesity are uterine and oesophageal cancer.^6^ In the present analysis, a trend toward a reduction of oesophageal cancer was observed with GLP‐1RA, although differences between treatment arms did not reach statistical significance. This is not surprising, considering the relatively low incidence and therefore the small number of observed cases of oesophageal cancer. The limited sample size and the insufficient duration of exposure could well explain the lack of significant results for all the other obesity‐related cancers, despite the relevant weight loss induced by GLP‐1RA. This could also explain the discrepancy of the present results from those of observational studies.^21^, ^28^

The increase in the number of reported cases of colorectal cancer in patients allocated to GLP‐1RA is surprising since the weight loss induced by GLP‐1RA should reduce the risk of this malignancy.^59^ Interestingly, this apparent detrimental effect of treatment is evident in shorter‐term but not in longer‐term trials. The results of such post hoc subgroup analysis should be interpreted very cautiously, considering that the limited number of observed cases prevents the detection of significant differences between subgroups of trials. However, it can be speculated that side effects of GLP‐1RA treatment, such as anorexia, nausea, vomiting, constipation and diarrhoea, induce physicians to perform diagnostic procedures that reveal underlying (and pre‐existing) tumours; this phenomenon would produce an apparent increase in incident cases at the beginning of treatment, which fades in the longer term. This highlights a major limitation of the present study: in the included RCTs, malignancies were not enlisted among pre‐specified endpoints; therefore, they were investigated and subsequently reported as SAEs only when symptoms referred by patients suggested the possibility of a malignancy, with the possibility of underdiagnosis of incident cases in the control arm or of misclassifying as incident cases pre‐existing malignancies in the active treatment arm. On the other hand, a direct effect of GLP‐1RA on colon oncogenesis may have some biological plausibility: GLP‐1 receptor expression has been detected in rodents and human colorectal epithelial tumours, even though reduced in comparison with adjacent healthy colon mucosa samples^60^; in rodents, it has also been proposed that GLP‐1 receptor signalling may regulate the expansion of colon mucosa through fibroblast growth factor 7,^60^ although an effect of GLP‐1RA on tumorigenesis has been demonstrated only in mice distal small intestine and not in colon mucosa.^60^, ^61^ On the other hand, GLP‐1 seems to exert an anti‐inflammatory effect on the mucosa of mice through increased antioxidant enzymes and reduced expression of transforming growth factor‐1, phosphatidylinositol‐3‐kinase and inflammatory cytokines such as nuclear factor kappa B, interleukin‐6 and interferon‐γ.^62^ Therefore, data from preclinical studies may either support a beneficial or detrimental effect of GLP‐1RA on colon tumorigenesis.

Another clear safety signal is related to the incidence of thyroid cancer. The present analysis is in line, on a larger data set, with our previous report of an increased incidence of thyroid cancer, which is more evident in longer‐term trials.^29^ This is not surprising, since the previous result was obtained on a fraction of the present dataset. In contrast with previous assertions,^29^ the association of GLP‐1RA with thyroid cancer retains statistical significance even after the inclusion of SELECT^33^ and other recent trials. This finding is in contrast with expectations for anti‐obesity agents, since the incidence of thyroid malignancies is expected to be reduced by weight loss.^6^ A specific effect of GLP‐1RAs on the thyroid gland may also have a biological plausibility, because GLP‐1 receptor overexpression has been observed in differentiated thyroid tumours cells, in respect to normal thyrocytes,^63^ although no net effect of GLP‐1 receptors on these cells has been demonstrated.^63^, ^64^, ^65^ Further mechanistic studies are needed to reach definitive conclusions on the biological plausibility of the association of GLP‐1 receptor stimulation with thyroid cancer. Epidemiologic studies also provided conflicting results on this point.^21^, ^27^, ^30^, ^31^ The results of observational studies on the association of GLP‐1RA with thyroid cancer should be interpreted cautiously: on one hand, the warnings of regulatory authorities on medullary thyroid cancer could lead some clinicians to over‐investigate thyroid nodules, with an increased chance of diagnosing thyroid cancer; on the other hand, the same warnings could lead some clinicians to avoid these drugs in all patients with thyroid nodules. The present meta‐analysis, performed on randomized trials only, overcomes the latter limitation. In addition, the sensitivity analysis confirming the association in double‐blind trials suggests that this result is independent of any detection bias.

Regarding preclinical studies, a clearer insight into the possible direct effect of GLP1‐RA on thyroid and colon tumorigenesis may be provided by recent machine learning‐enabled computational methods,^66^, ^67^ which may enhance the prediction of possible expression patterns and signalling pathways of GLP‐1 receptor in thyroid and colon normal and cancer tissues.

Considering the relatively low incidence of thyroid cancer, even an increase of relative risk would determine a limited increase in absolute risk, with a number needed to harm over 1300.^29^ This possible detrimental effect of GLP‐1RA on the incidence of thyroid cancer, if confirmed, should therefore be considered in the wider context of proven benefits of therapy, such as prevention of cardiovascular disease,^33^ prevention^33^ or improvement^68^, ^69^ of type 2 diabetes and possibly other obesity‐related complications.^70^

We already highlighted some limitations of the present study, that is, the limited sample size and study duration. In fact, the small number of recorded events for many malignancies could have prevented the observation of either beneficial or detrimental effects. Furthermore, the small number of recorded events prevented subgroup analyses for individual molecules of the class for each specific malignancy, and separate analyses for different doses for each available drug; this could be relevant, as different drugs have different effects on body weight, and weight reduction is dose‐dependent.^71^ In addition, the length of exposure to GLP‐1RA could have been insufficient to detect some longer‐term effects, increasing the chance of falsely negative results. Notably, many observational studies could have the advantage of a greater sample size, but they also usually suffer from a relatively short length of drug exposure. The fact that incident malignancies were not pre‐specified endpoints, and that cases were not formally adjudicated, is another major limitation, leading to the possibility of undetected cases and/or misdiagnosis, as already stated before. Unfortunately, a randomized trial on diabetes or obesity with cancer as a primary endpoint would be scarcely feasible, and observational studies suffer from the very same issues of underdiagnosis and misdiagnosis. Some further limitations of the present meta‐analysis should be considered for a correct interpretation of its results. The analysis was performed considering the overall number of incident cases, without any information on the date of onset; therefore, it was not possible to exclude early‐onset (and probably pre‐existing) malignancies, reported soon after the beginning of treatment. The subgroup analysis of shorter‐and longer‐term trials only partly surrogates the actual analysis based on time to event in individual patients. A further limitation is the specificity of the case mix: the population enrolled in clinical trials does not fully represent those receiving treatment in routine clinical practice. In particular, study protocols of most trials exclude patients at higher risk of medullary thyroid cancer. In addition, although most large‐scale trials included were performed versus placebo, the meta‐analysis also collected trials in which GLP‐1RA were compared with other active drugs; such comparators could theoretically be associated with either an increase or a reduction in the risk of certain cancers, possibly affecting results. Furthermore, it was not possible to retrieve data on the incidence of cancer from one large trial with liraglutide,^68^ although the analysis of funnel plots did not suggest selective reporting. Interestingly, analysis from this trial showed that the 5‐year cancer mortality in those with liraglutide is not different from comparators.^72^

On the other hand, some strengths of the present analysis should be considered. In clinical trials, the allocation to different treatments is determined by randomization. This warrants the comparability between patients on GLP‐1RA and control arms, thus overcoming the major limitation in observational studies, which may be affected by uncontrolled (or inadequately controlled) confounders. Moreover, in this meta‐analysis, the heterogeneity in results between studies is very low, thus further reducing the possibility of confounding factors. Notably, statistical heterogeneity was low despite the fact that different molecules of the class have different efficacy as weight‐reducing agents.^71^, ^73^ Another strength is represented by the retrieval of serious adverse events from multiple sources, that is, public trial repositories, and not only publications; this limits the possibility of publication bias.

The results of the present meta‐analysis are partly discordant from those of observational studies. Longer‐term observations on cohorts of patients treated with GLP‐1RA reported significant reductions in the risk of some obesity‐related malignancies, such as prostate^28^, ^74^ and colorectal^21^, ^28^ cancer, which were not detected in clinical trials. The discrepancy could depend on differences in case mix and/or shorter duration of observation in clinical trials, but it could also be the effects of unaccounted confounding bias in observational studies. Notably, results of clinical trials are compatible with the reported observations of a reduction in the risk of uterine^28^ and oesophageal^75^ cancer. The increase in the risk of kidney cancer in comparison with metformin, reported by one observational study^21^ was not confirmed in the present meta‐analysis, but the number of observed events is too small to draw any conclusion. The results of observational studies on the association of GLP1‐RA with thyroid cancer are heterogeneous, reporting either an increased^27^, ^28^, ^31^ or unmodified^21^, ^30^ incidence. The difference in results of clinical trials could be due either to a different case mix or to possible prescription bias: since the summary of product characteristics in the US and Europe,^23^, ^24^ on the basis of preclinical studies only, warns of possible risks in patients with a personal or familial history of medullary carcinoma, physicians could be less prone to prescribe a GLP‐1RA in patients with thyroid nodules, which are often not reported in large databases used for retrospective observational studies.

In conclusion, in randomized clinical trials available to date, GLP‐1RA do not appear to produce major effects on most malignancies, either beneficial or detrimental; however, the results of this meta‐analysis should be considered hypothesis‐generating rather than conclusive. A reduction of the risk of those malignancies that are more closely related to obesity, such as uterine and oesophageal cancers, seems possible; however, further data are needed to draw clear conclusions on this point. In this respect, observational studies, which are inevitably hampered by confounding bias, can be confirmatory of evidence from randomized trials. Conversely, very long‐term trials with malignancies as the principal outcome are hardly feasible. Results of ongoing large‐scale trials on GLP‐1RA and other molecules that also act as GLP‐1R agonists (e.g., dual GLP‐1/GIP and GLP‐1/glucagon agonists) will add further data, which will need to be incorporated in future meta‐analyses on this issue. On the other hand, a moderate increase in the incidence of colorectal cancer, which is more evident in shorter‐term trials, could be the effect of over‐diagnosis induced by the side effects of GLP‐1RA; however, this issue deserves further investigation through specific mechanistic studies and long‐term observational studies. Finally, the possibility of a moderate increase in the risk of thyroid cancer, particularly in longer‐term trials, cannot be ruled out, and it needs to be clarified because of its relevance in the assessment of the risk/benefit ratio of GLP‐1RA treatment in individual patients.

## AUTHOR CONTRIBUTIONS

G.A.S and E.M. made the analysis plan, researched data, performed analyses, contributed to the discussion and wrote the first draft of the manuscript. C.M., G.G.D.V., C.B. and M.M. contributed to data research and reviewed and edited the manuscript. All the authors had full access to study data, approved the final version of the manuscript and took responsibility for data integrity and analysis accuracy.

## CONFLICT OF INTEREST STATEMENT

GAS has received speaking or consultancy fees from Astra Zeneca, Eli‐Lilly and Novo Nordisk, outside the submitted work. MM has received speaking fees from Astra Zeneca, Boehringer‐Ingelheim, Eli‐Lilly, Merck, Novo Nordisk and Sanofi, outside the submitted work. The unit directed by EM has received research grants from Abbott, Eli‐Lilly and Novo Nordisk, outside the submitted work. EM has received consultancy fees or speaking fees from Astra Zeneca, Bayer, Boehringer‐Ingelheim, Coresearch, Dexcom, Eli‐Lilly, Molteni, Novo Nordisk, Pidkare and Sanofi, outside the submitted work. C.M., G.G.D.V. and C.B. do not have any competing interests to disclose.

## PEER REVIEW

The peer review history for this article is available at https://www.webofscience.com/api/gateway/wos/peer‐review/10.1111/dom.16489.

## Supporting information


**Data S1.** Supporting information.

## Data Availability

Data sharing not applicable—no new data generated, or the article describes entirely theoretical research.
