# Supplementary material for: GLP‐1 receptor agonists and the risk for cancer: A meta‐analysis of randomized controlled trials
Source: Diabetes Obes Metab. 2025 May 29;27(8):4454–68. doi: 10.1111/dom.16489 (PMC12232360; doi:10.1111/dom.16489)
Supplement: Supplementary file 1 — Data S1. Supporting information. [file DOM-27-4454-s001.pdf]

GLP1-Receptor Agonists and the risk for cancer: a meta-analysis of randomized controlled trials

**PUBMED :** ("exenatide"[MeSH Terms] OR "exenatide"[All Fields]) OR ("liraglutide"[MeSH Terms] OR "liraglutide"[All Fields]) OR ("lixisenatide"[Supplementary Concept] OR "lixisenatide"[All Fields]) OR ("rGLP-1 protein"[Supplementary Concept] OR "rGLP-1 protein"[All Fields] OR "albiglutide"[All Fields]) OR ("dulaglutide"[Supplementary Concept] OR "dulaglutide"[All Fields]) OR ("semaglutide"[Supplementary Concept] OR "semaglutide"[All Fields]) AND (Randomized Controlled Trial[ptyp] AND "humans"[MeSH Terms])
**Clinicaltrials.gov:** (exenatide OR liraglutide OR lixisenatide OR dulaglutide, OR semaglutide) Active, not recruiting, Completed, Terminated Studies | Interventional Studies.
**Cochrane database** exenatide or liraglutide or lixisenatide or dulaglutide or semaglutide in Title Abstract Keyword - (Word variations have been searched)
**Embase** ('exenatide'/exp OR 'exenatide' OR 'liraglutide'/exp OR 'liraglutide' OR 'lixisenatide'/exp OR 'lixisenatide' OR 'rglp-1 protein' OR 'semaglutide'/exp OR 'semaglutide' OR 'dulaglutide'/exp OR 'dulaglutide') AND ('(randomized controlled trial)'/exp OR '(randomized controlled trial)') AND ('human'/exp OR 'human'**)**

Table S1 – Information on search string

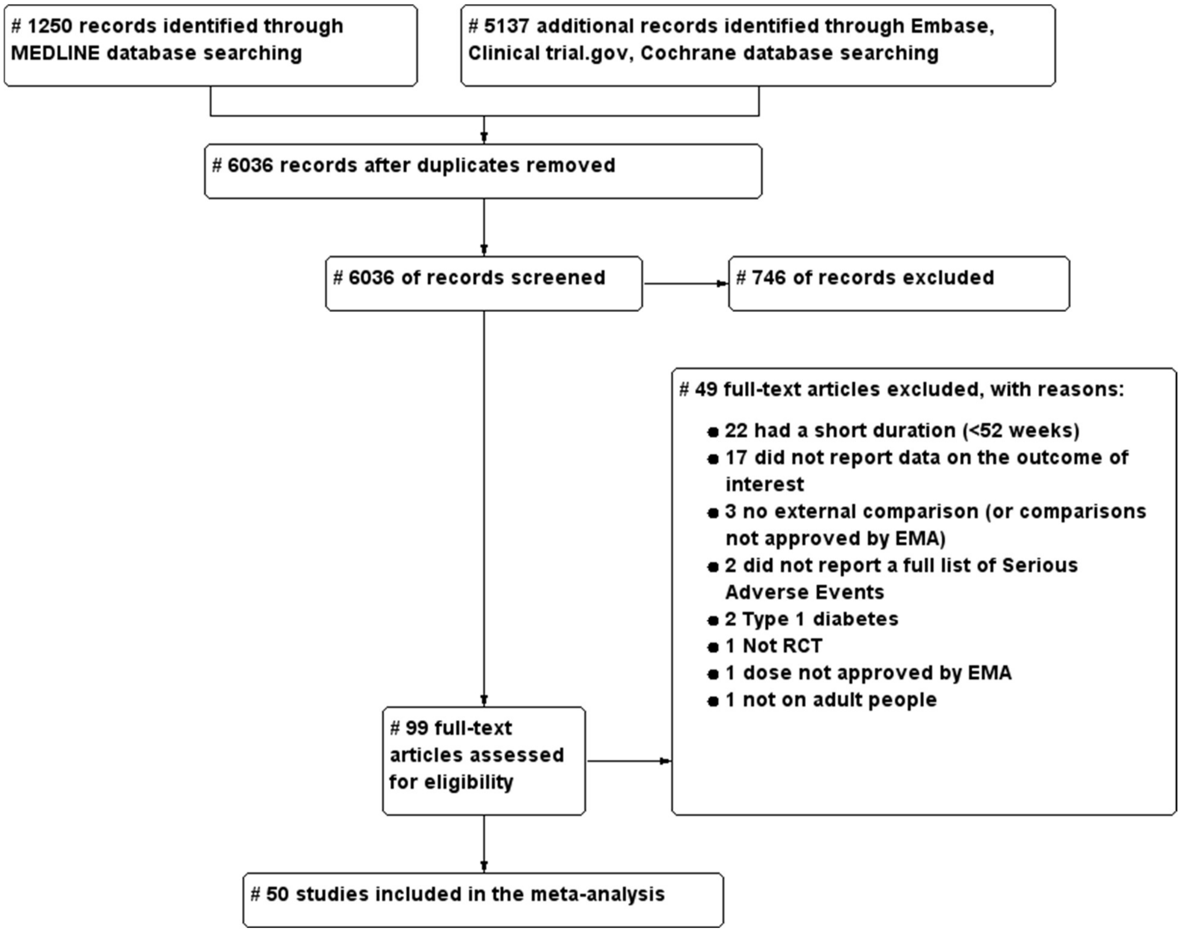

Figure S1: trial flow summary.

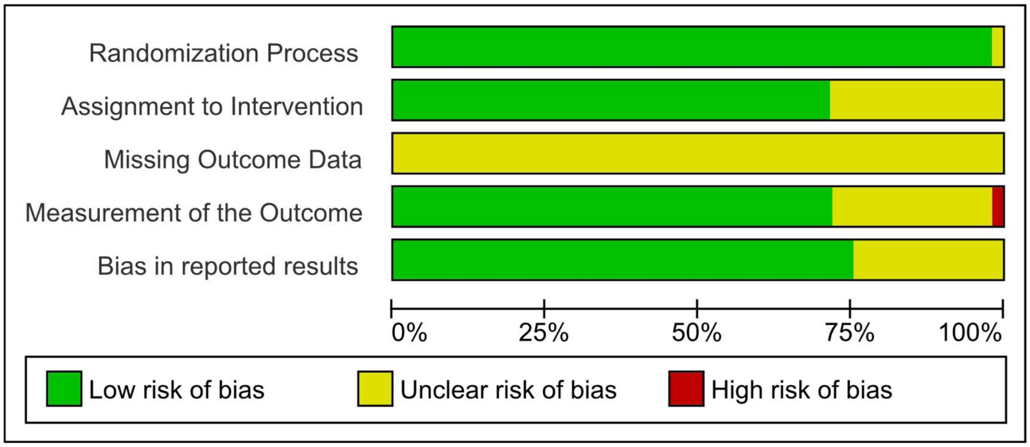

Figure S2: *Risk of bias graph*

Study	Randomization Process	Assignment to Intervention	Missing Outcome Data	Measurement of the Outcome	Bias in reported results
Ahren 2017	+	+	?	+	+
Aroda 2019	+	?	?	+	+
Astrup 2013	+	?	?	?	+
Blonde 2015	?	+	?	?	?
Buse 2020	+	?	?	?	?
Davies 2015	+	+	?	+	?
Davies 2021	+	+	?	?	?
Gallwitz 2012	+	?	?	?	+
Garber 2011	+	+	?	?	+
Garvey 2020	+	?	?	?	?
Garvey 2022	+	+	?	+	+
Gerstein 2019	+	+	?	+	+
Giorgino 2015	+	?	?	+	+
Gough 2015	+	?	?	+	+
Holman 2017	+	+	?	+	+
Husain 2019	+	+	?	+	+
Jaiswal 2015	+	?	?	−	?
Kadowaki 2022	+	+	?	+	+
Kaku 2018	+	+	?	?	?
Knop 2023	+	+	?	+	+
Kosiborod 2023	+	+	?	+	+
Lincoff 2023	+	+	?	+	?
Marso 2016	+	+	?	+	+
Marso 2016 a	+	+	?	+	+
Miyagawa 2015	+	+	?	+	+
Nahra 2022	+	+	?	+	+
Nauck 2007	+	+	?	+	+
Nauck 2013	+	+	?	+	+
O' Neill 2018	+	+	?	+	?
Perkovic 2024	+	?	?	+	?
Pfeffer 2015	+	+	?	+	+
Pi-Sunyer 2015	+	+	?	?	?
Pratley 2011	+	+	?	+	+
Pratley 2019	+	+	?	+	+
Rodbard 2019	+	+	?	+	+
Rosenstock 2019	+	+	?	+	+
Rubino 2022	+	+	?	+	+
Ruff 2021	+	+	?	+	+
Tuttle 2018	+	?	?	?	+
Umpierrez 2014	+	?	?	+	+
Unger 2022	+	?	?	?	?
Wadden 2013	+	+	?	+	+
Wadden 2020	+	+	?	+	+
Wadden 2021	+	+	?	+	+
Wang 2019	+	?	?	?	+
Weinstock 2015	+	?	?	+	+
Wilding 2021	+	+	?	+	+
Yamada 2020	+	+	?	?	+
Zinman 2019	+	+	?	+	+

Figure S3: *Risk of bias summary*

#	Trial name	Reason for exclusion
1	Armstrong2020	Less than 52 weeks
2	Silver 2023	Less than 52 weeks
3	Mashayekhi 2023	Less than 52 weeks
4	Galindo 2023	Less than 52 weeks
5	Kelly 2020	Minor than 18 years old
6	Armstrong 2016	Less than 52 weeks
7	Cape Horn 2019	No comparator
8	Pratley 2019	Less than 52 weeks
9	Hirsch 2022	Less than 52 weeks
10	Neland 2021	Less than 52 weeks
11	Blackman 2016	Less than 52 weeks
12	Mathieux 2016	Type 1 diabetes
13	Billings 2018	Less than 52 weeks
14	Dejgaard 2017	Type 1 diabetes
15	Russell-jones 2009	Less than 52 weeks
16	Rizzo 2016	Nonrandomized CT
17	Lind 2015	Less than 52 weeks
18	Lee 2014	Less than 52 weeks
19	Mathieux 2014	Less than 52 weeks
20	Holst 2015	Less than 52 weeks
21	Davies 2017	Less than 52 weeks
22	Ghusn 2022	Less than 52 weeks
23	Newsome 2021	Lower dose
24	Aroda 2023	No comparator
25	Ahmann 2018	No comparator
26	Eckard 2024	Less than 52 weeks
27	The Grade Study research group, 2022	No available results
28	Knop 2023	No available results
29	Mu 2024	Less than 52 weeks
30	Loomba 2023	Less than 52 weeks
31	Furusawa 2023	Less than 52 weeks
32	Dwibedi 2024	Less than 52 weeks
33	Bunck 2009	Malignancies not reported
34	Derosa 2010	Malignancies not reported
35	Derosa 2011	Malignancies not reported
36	Diamant 2014	Malignancies not reported
37	Gudbergsen 2021	Malignancies not reported
38	Iepsen2015	Malignancies not reported
39	Ikonomidis 2020	Malignancies not reported
40	Inagaki 2012	Malignancies not reported
41	Jabbour 2020	Malignancies not reported
42	Kaku 2019	Malignancies not reported
43	Lingvay 2019	Malignancies not reported
44	Lundgren 2021	Malignancies not reported
45	Samson 2011	Malignancies not reported
46	Tronieri 2020	Malignancies not reported
47	Wang 2019 a	Malignancies not reported
48	Zhang 2020	Malignancies not reported
49	Kosiborod 2024	Malignancies not reported

Table S2: list of excluded trials, with reasons for exclusion.

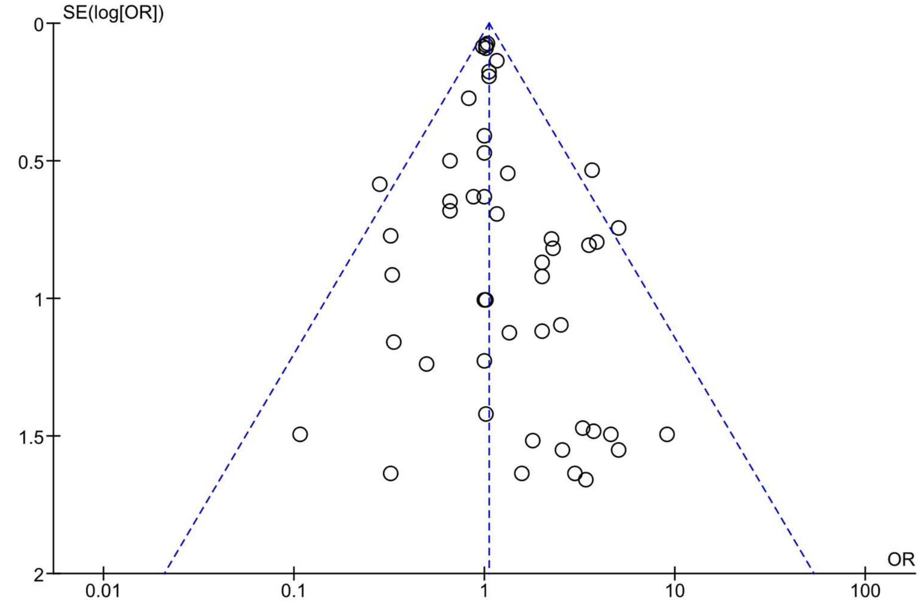

Figure S4: Funnel plot (Risk for overall cancer)

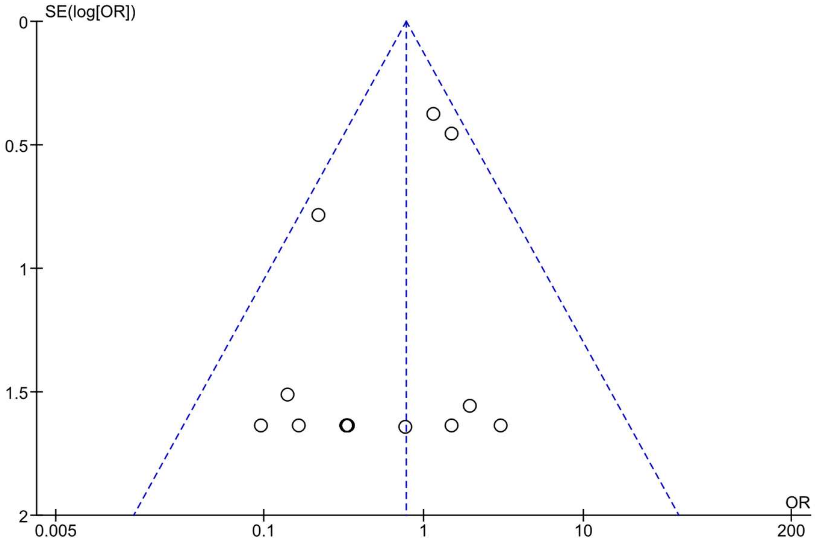

Figure S5: Funnel plot (Risk for uterine cancer)

Study or Subgroup	GLP-1RA Events	GLP-1RA Total	Control Events	Control Total	Weight	Odds Ratio M-H, Fixed, 95% CI	Risk of Bias A	B	C	D	E
Aroda 2019	0	506	1	506	6.4%	0.33 [0.01, 8.19]	+	?	?	+	+
Gerstein 2019	6	4949	6	4952	25.4%	1.00 [0.32, 3.10]	+	+	?	+	+
Holman 2017	3	7356	6	7396	25.4%	0.50 [0.13, 2.01]	+	+	?	+	+
Kaku 2018	1	480	0	121	3.4%	0.76 [0.03, 18.78]	+	+	?	?	?
Lincoff 2023	1	8803	1	8801	4.2%	1.00 [0.06, 15.99]	+	+	?	+	?
Marso 2016 a	4	1648	6	1649	25.4%	0.67 [0.19, 2.37]	+	+	?	+	+
Weinstock 2015	0	606	1	177	9.8%	0.10 [0.00, 2.39]	+	?	?	+	+
**Total (95% CI)**		**24348**		**23602**	**100.0%**	**0.65 [0.34, 1.23]**					
Total events	15		21								

Heterogeneity: Chi² = 2.31, df = 6 (P = 0.89); $I^2 = 0\%$

Test for overall effect: Z = 1.32 (P = 0.19)

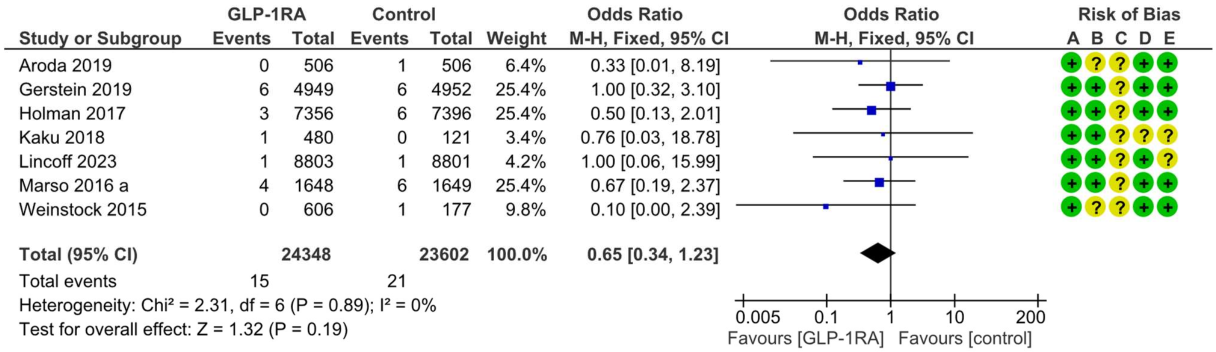

Risk of bias legend

(**A**) Randomization Process

(**B**) Assignment to Intervention

(**C**) Missing Outcome Data

(**D**) Measurement of the Outcome

(**E**) Bias in reported results

Fig. S6: Difference in risk for esophageal cancer between GLP-1RA and comparators

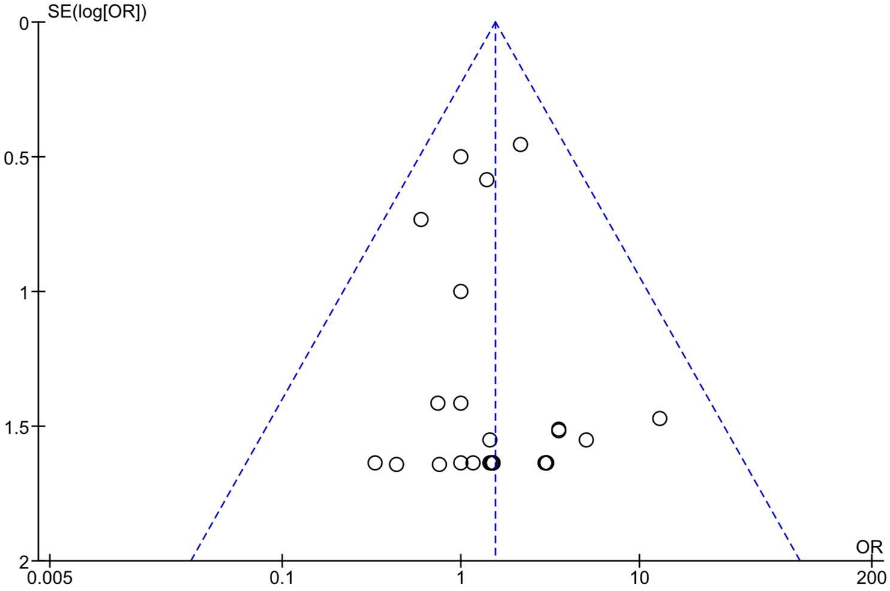

Figure S7: Funnel plot (Risk for thyroid cancer)

Study or Subgroup	GLP-1RA Events	GLP-1RA Total	Control Events	Control Total	Weight	Odds Ratio M-H, Fixed, 95% CI
**10.53.1 Shorter than 104 weeks**						
Ahren 2017	1	818	0	407	1.5%	1.50 [0.06, 36.79]
Buse 2020	1	253	0	98	1.6%	1.17 [0.05, 28.97]
Davies 2015	0	211	1	212	3.4%	0.33 [0.01, 8.23]
Garber 2011	3	498	0	248	1.5%	3.51 [0.18, 68.23]
Gough 2015	1	414	0	413	1.1%	3.00 [0.12, 73.86]
Husain 2019	2	1591	0	1592	1.1%	5.01 [0.24, 104.43]
O' Neill 2018	0	103	1	136	2.9%	0.44 [0.02, 10.82]
Pratley 2011	1	439	0	219	1.5%	1.50 [0.06, 37.01]
Pratley 2019	1	284	0	142	1.5%	1.51 [0.06, 37.25]
Ruff 2021	3	2074	5	2070	11.4%	0.60 [0.14, 2.51]
Wadden 2013	1	212	0	210	1.1%	2.99 [0.12, 73.71]
Wadden 2020	1	142	0	140	1.1%	2.98 [0.12, 73.75]
Wadden 2021	1	407	0	204	1.5%	1.51 [0.06, 37.21]
Wang 2019	1	515	0	259	1.5%	1.51 [0.06, 37.27]
Wilding 2021	1	1306	0	655	1.5%	1.51 [0.06, 37.03]
Yamada 2020	1	195	0	49	1.8%	0.76 [0.03, 19.03]
**Subtotal (95% CI)**		**9462**		**7054**	**36.3%**	**1.31 [0.68, 2.52]**
Total events	19		7			
Heterogeneity: Chi² = 4.39, df = 15 (P = 1.00); I² = 0%						
Test for overall effect: Z = 0.79 (P = 0.43)						
**10.53.2 104 weeks or longer**						
Gerstein 2019	6	4949	0	4952	1.1%	13.02 [0.73, 231.25]
Giorgino 2015	1	545	0	262	1.5%	1.45 [0.06, 35.62]
Holman 2017	15	7356	7	7396	15.9%	2.16 [0.88, 5.29]
Lincoff 2023	7	8803	5	8801	11.4%	1.40 [0.44, 4.41]
Marso 2016	2	4668	2	4672	4.6%	1.00 [0.14, 7.11]
Marso 2016 a	8	1648	8	1649	18.2%	1.00 [0.37, 2.67]
Nauck 2013	1	483	1	363	2.6%	0.75 [0.05, 12.05]
Pfeffer 2015	1	3034	1	3034	2.3%	1.00 [0.06, 15.99]
Pi-Sunyer 2015	3	2481	0	1242	1.5%	3.51 [0.18, 67.99]
Rosenstock 2019	1	1397	0	467	1.7%	1.00 [0.04, 24.70]
Unger 2022	1	980	0	984	1.1%	3.02 [0.12, 74.11]
Weinstock 2015	2	606	0	177	1.8%	1.47 [0.07, 30.72]
**Subtotal (95% CI)**		**36950**		**33999**	**63.7%**	**1.68 [1.05, 2.70]**
Total events	48		24			
Heterogeneity: Chi² = 4.63, df = 11 (P = 0.95); I² = 0%						
Test for overall effect: Z = 2.17 (P = 0.03)						
**Total (95% CI)**		**46412**		**41053**	**100.0%**	**1.55 [1.05, 2.27]**
Total events	67		31			
Heterogeneity: Chi² = 9.23, df = 27 (P = 1.00); I² = 0%						
Test for overall effect: Z = 2.23 (P = 0.03)						
Test for subgroup differences: Chi² = 0.38, df = 1 (P = 0.54), I² = 0%						

Odds Ratio M-H, Fixed, 95% CI — Risk of Bias A B C D E

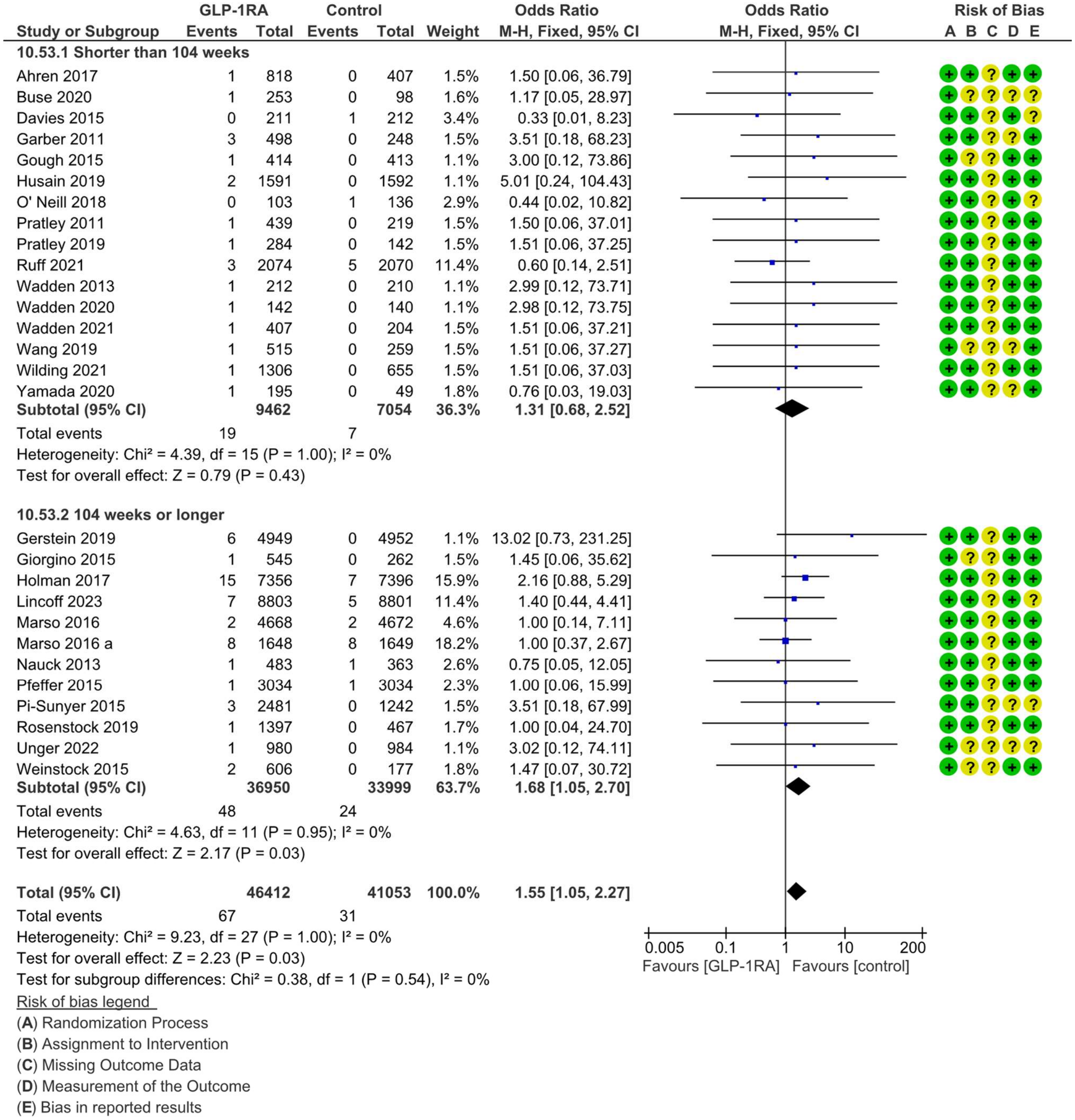

Risk of bias legend
(**A**) Randomization Process
(**B**) Assignment to Intervention
(**C**) Missing Outcome Data
(**D**) Measurement of the Outcome
(**E**) Bias in reported results

Fig. S8: Difference in risk for Thyroid cancer: subgroup analysis for different duration

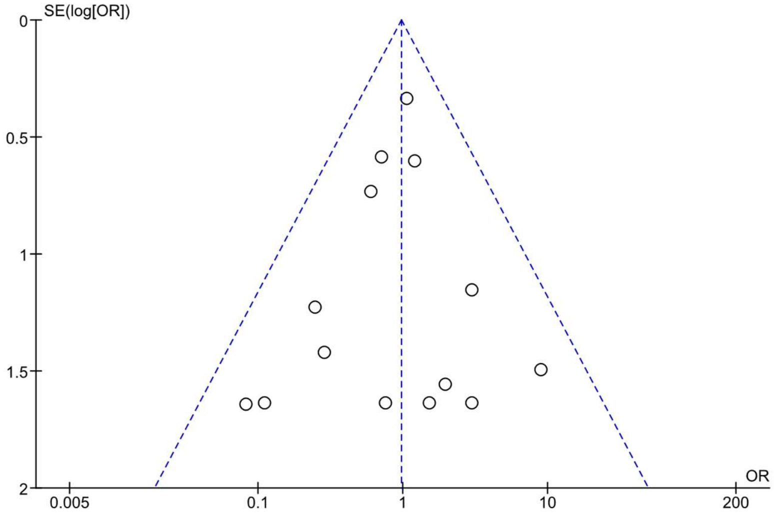

Figure S9: Funnel plot (Risk for colorectal cancer)

Study or Subgroup	GLP-1RA Events	GLP-1RA Total	Control Events	Control Total	Weight	Odds Ratio M-H, Fixed, 95% CI	A	B	C	D	E
**10.60.2 104 weeks or shorter**											
Blonde 2015	1	588	0	296	0.4%	1.51 [0.06, 37.28]	?	+	?	?	?
Buse 2020	4	253	0	98	0.5%	3.55 [0.19, 66.61]	+	?	?	?	?
Davies 2015	1	211	0	212	0.3%	3.03 [0.12, 74.76]	+	+	?	+	?
Davies 2021	1	805	0	402	0.4%	1.50 [0.06, 36.93]	+	+	?	?	?
Husain 2019	5	1591	2	1592	1.3%	2.51 [0.49, 12.94]	+	+	?	+	+
Kaku 2018	1	480	0	121	0.5%	0.76 [0.03, 18.78]	+	+	?	?	?
Knop 2023	2	334	0	333	0.3%	5.02 [0.24, 104.86]	+	+	?	+	+
Pi-Sunyer 2015	5	2481	0	1242	0.4%	5.52 [0.30, 99.89]	+	+	?	?	?
Pratley 2011	1	439	0	219	0.4%	1.50 [0.06, 37.01]	+	+	?	+	+
Rodbard 2019	1	410	0	409	0.3%	3.00 [0.12, 73.86]	+	+	?	+	+
Rosenstock 2019	2	1397	1	467	1.0%	0.67 [0.06, 7.38]	+	+	?	+	+
Zinman 2019	2	547	0	184	0.5%	1.69 [0.08, 35.39]	+	+	?	+	+
**Subtotal (95% CI)**		**9536**		**5575**	**6.5%**	**2.28 [1.04, 4.98]**					
Total events	26		3								
Heterogeneity: Chi² = 2.46, df = 11 (P = 1.00); I² = 0%											
Test for overall effect: Z = 2.06 (P = 0.04)											
**10.60.3 Longer than 104 weeks**											
Aroda 2019	1	506	0	506	0.3%	3.01 [0.12, 73.96]	+	?	?	+	+
Gallwitz 2012	3	490	3	487	2.0%	0.99 [0.20, 4.95]	+	?	?	?	+
Garber 2011	1	498	1	248	0.9%	0.50 [0.03, 7.98]	+	+	?	?	+
Gerstein 2019	40	4949	34	4952	22.1%	1.18 [0.74, 1.86]	+	+	?	+	+
Holman 2017	42	7356	32	7396	20.8%	1.32 [0.83, 2.10]	+	+	?	+	+
Lincoff 2023	35	8803	27	8801	17.6%	1.30 [0.78, 2.14]	+	+	?	+	?
Marso 2016	2	4668	8	4672	5.2%	0.25 [0.05, 1.18]	+	+	?	+	+
Marso 2016 a	33	1648	27	1649	17.3%	1.23 [0.73, 2.05]	+	+	?	+	+
Nauck 2013	1	483	0	363	0.4%	2.26 [0.09, 55.64]	+	+	?	+	+
Pfeffer 2015	12	3034	9	3034	5.9%	1.33 [0.56, 3.17]	+	+	?	+	+
Unger 2022	2	980	1	984	0.7%	2.01 [0.18, 22.21]	+	?	?	?	?
Weinstock 2015	1	606	0	177	0.5%	0.88 [0.04, 21.68]	+	?	?	+	+
**Subtotal (95% CI)**		**34021**		**33269**	**93.5%**	**1.20 [0.96, 1.50]**					
Total events	173		142								
Heterogeneity: Chi² = 5.38, df = 11 (P = 0.91); I² = 0%											
Test for overall effect: Z = 1.64 (P = 0.10)											
**Total (95% CI)**		**43557**		**38844**	**100.0%**	**1.27 [1.03, 1.57]**					
Total events	199		145								
Heterogeneity: Chi² = 9.42, df = 23 (P = 0.99); I² = 0%											
Test for overall effect: Z = 2.24 (P = 0.03)											
Test for subgroup differences: Chi² = 2.36, df = 1 (P = 0.12), I² = 57.7%											

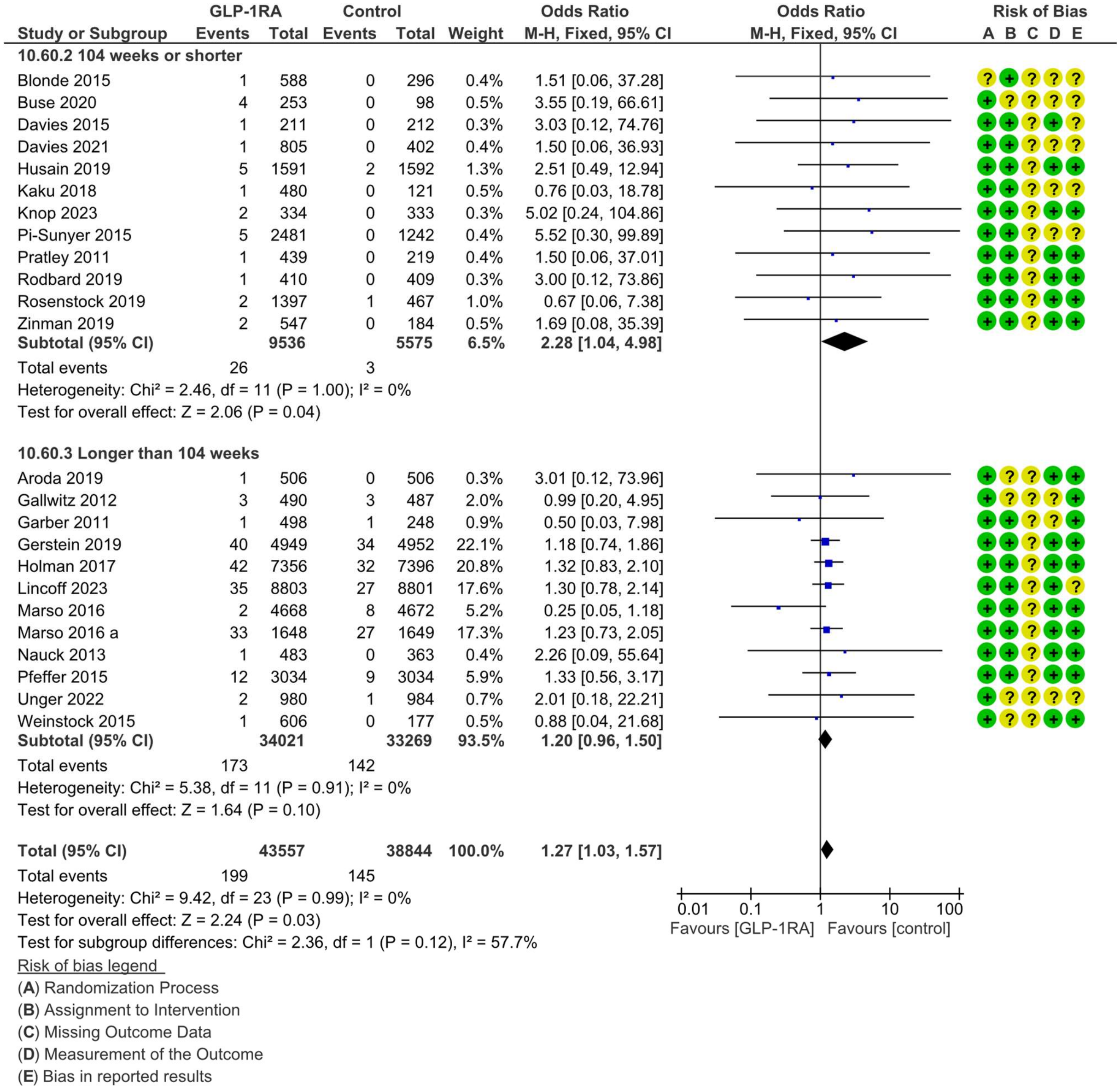

Risk of bias legend
(**A**) Randomization Process
(**B**) Assignment to Intervention
(**C**) Missing Outcome Data
(**D**) Measurement of the Outcome
(**E**) Bias in reported results

Fig. S10: Difference in risk for colon cancer: subgroup analysis for different duration of the study

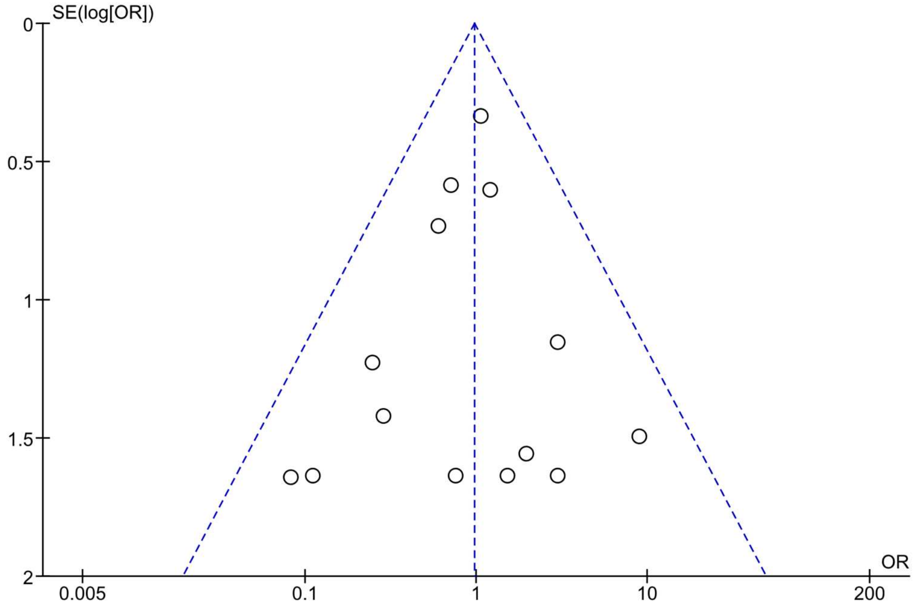

Figure S11: Funnel plot (Risk for gastric cancer)

Study or Subgroup	GLP-1RA Events	GLP-1RA Total	Control Events	Control Total	Weight	Odds Ratio M-H, Fixed, 95% CI	Risk of Bias A	B	C	D	E
Buse 2020	2	253	0	98	1.5%	1.96 [0.09, 41.15]	+	?	?	?	?
Davies 2021	1	805	2	402	5.7%	0.25 [0.02, 2.75]	+	+	?	?	?
Gerstein 2019	18	4949	17	4952	36.1%	1.06 [0.55, 2.06]	+	+	?	+	+
Holman 2017	5	7356	7	7396	14.9%	0.72 [0.23, 2.26]	+	+	?	+	+
Husain 2019	1	1591	0	1592	1.1%	3.00 [0.12, 73.79]	+	+	?	+	+
Kaku 2018	1	480	0	121	1.7%	0.76 [0.03, 18.78]	+	+	?	?	?
Lincoff 2023	6	8803	5	8801	10.7%	1.20 [0.37, 3.93]	+	+	?	+	?
Marso 2016	4	4668	0	4672	1.1%	9.02 [0.49, 167.50]	+	+	?	+	+
Marso 2016 a	3	1648	5	1649	10.6%	0.60 [0.14, 2.51]	+	+	?	+	+
Pfeffer 2015	3	3034	1	3034	2.1%	3.00 [0.31, 28.88]	+	+	?	+	+
Rosenstock 2019	0	1397	1	467	4.8%	0.11 [0.00, 2.74]	+	+	?	+	+
Tuttle 2018	1	383	0	194	1.4%	1.53 [0.06, 37.62]	+	?	?	?	+
Weinstock 2015	1	606	1	177	3.3%	0.29 [0.02, 4.67]	+	?	?	+	+
Yamada 2020	0	195	1	49	5.1%	0.08 [0.00, 2.06]	+	+	?	?	+
**Total (95% CI)**		**36168**		**33604**	**100.0%**	**0.97 [0.65, 1.46]**					
Total events	46		40								

Heterogeneity: Chi² = 10.83, df = 13 (P = 0.63); I² = 0%
Test for overall effect: Z = 0.15 (P = 0.88)

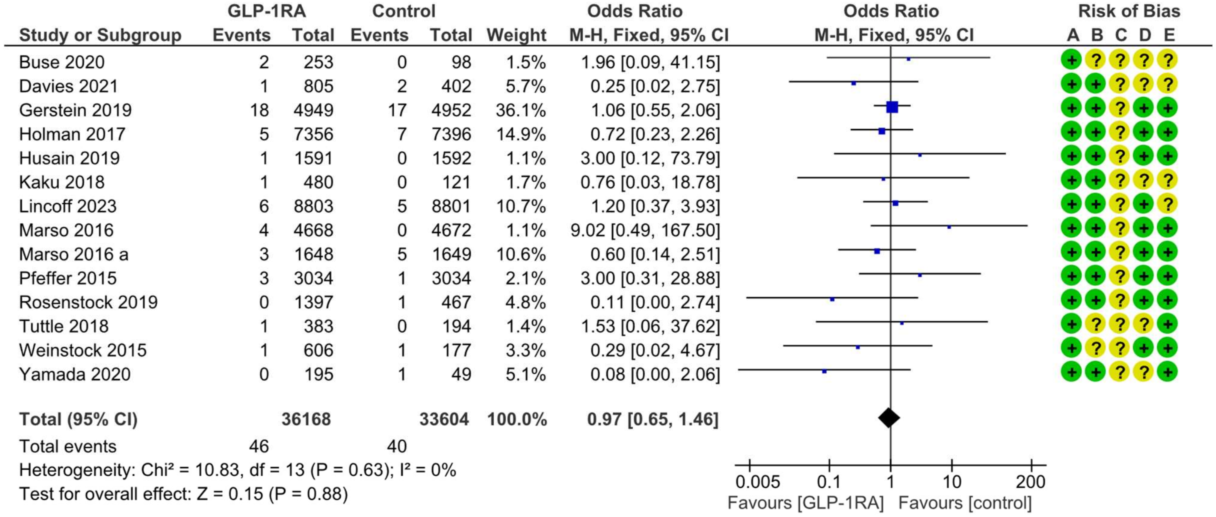

Risk of bias legend
(**A**) Randomization Process
(**B**) Assignment to Intervention
(**C**) Missing Outcome Data
(**D**) Measurement of the Outcome
(**E**) Bias in reported results

Fig. S12: Difference in risk for gastric cancer

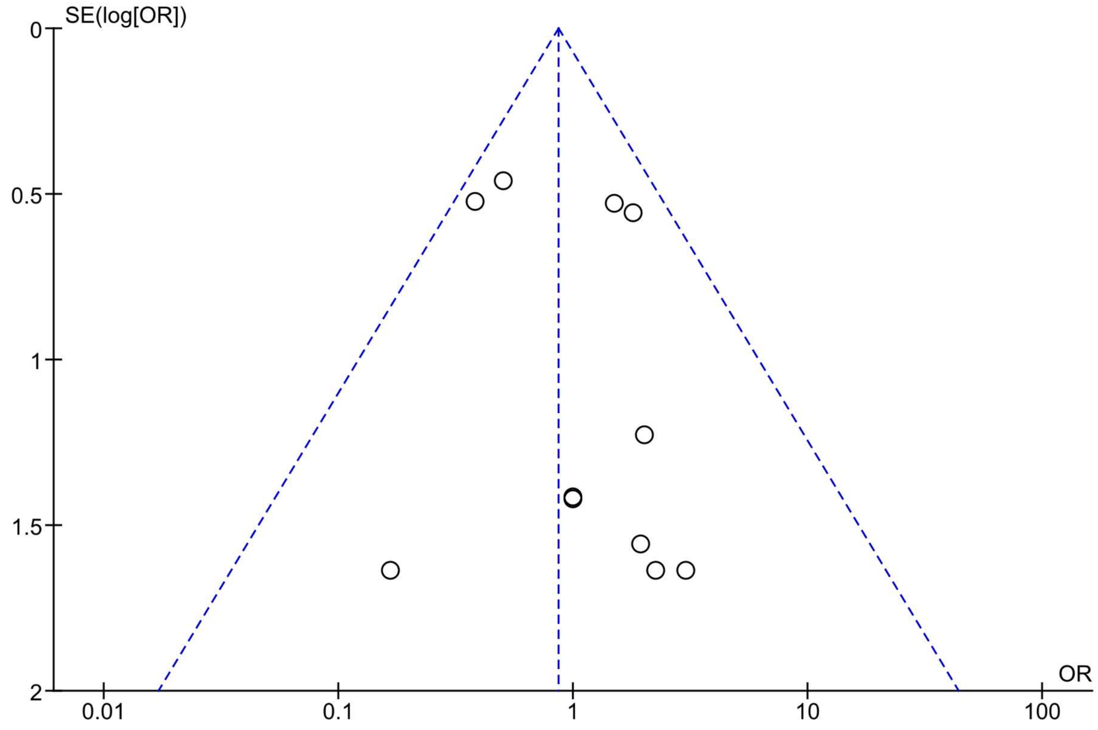

Figure S13: Funnel plot (Risk for liver cancer)

Study or Subgroup	GLP-1RA Events	GLP-1RA Total	Control Events	Control Total	Weight	Odds Ratio M-H, Fixed, 95% CI	Odds Ratio M-H, Fixed, 95% CI	Risk of Bias A	B	C	D	E
Buse 2020	2	253	0	98	1.6%	1.96 [0.09, 41.15]		+	?	?	?	?
Davies 2015	1	211	1	212	2.2%	1.00 [0.06, 16.17]		+	+	?	+	?
Davies 2021	0	805	1	402	4.5%	0.17 [0.01, 4.09]		+	+	?	?	?
Gerstein 2019	5	4949	13	4952	29.1%	0.38 [0.14, 1.08]		+	+	?	+	+
Holman 2017	7	7356	14	7396	31.2%	0.50 [0.20, 1.25]		+	+	?	+	+
Knop 2023	1	334	0	333	1.1%	3.00 [0.12, 73.91]		+	+	?	+	+
Lincoff 2023	9	8803	5	8801	11.2%	1.80 [0.60, 5.37]		+	+	?	+	?
Marso 2016	1	4668	1	4672	2.2%	1.00 [0.06, 16.01]		+	+	?	+	+
Marso 2016 a	9	1648	6	1649	13.4%	1.50 [0.53, 4.23]		+	+	?	+	+
Nauck 2013	1	483	0	363	1.3%	2.26 [0.09, 55.64]		+	+	?	+	+
Pfeffer 2015	2	3034	1	3034	2.2%	2.00 [0.18, 22.08]		+	+	?	+	+
**Total (95% CI)**		**32544**		**31912**	**100.0%**	**0.86 [0.56, 1.32]**						
Total events	38		42									

Heterogeneity: Chi² = 9.29, df = 10 (P = 0.51); I² = 0%

Test for overall effect: Z = 0.68 (P = 0.50)

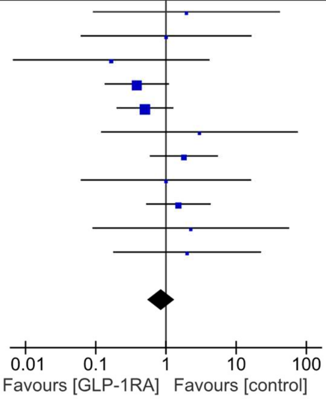

Risk of bias legend
(**A**) Randomization Process
(**B**) Assignment to Intervention
(**C**) Missing Outcome Data
(**D**) Measurement of the Outcome
(**E**) Bias in reported results

Fig. S14: Difference in risk for Liver cancer between GLP1- RA and control

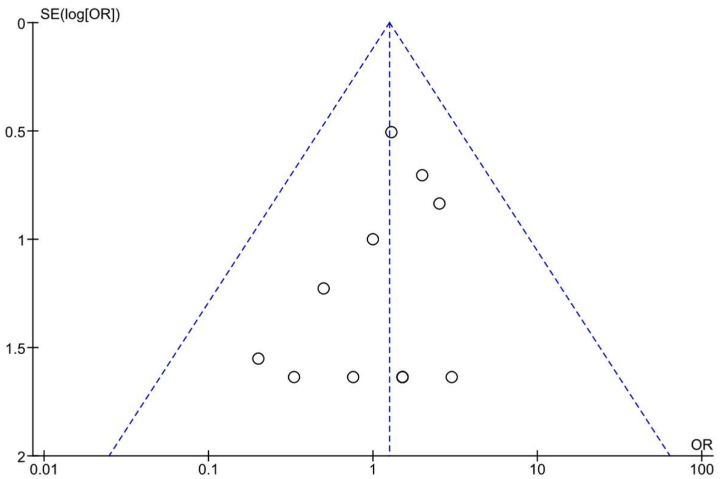

Fig. S15: Funnel plot (Difference in risk for Gallbladder cancer and Cholangiocarcinoma)

Study or Subgroup	GLP-1RA Events	GLP-1RA Total	Control Events	Control Total	Weight	Odds Ratio M-H, Fixed, 95% CI	Risk of Bias A	B	C	D	E
Blonde 2015	1	588	0	296	2.9%	1.51 [0.06, 37.28]	?	+	?	?	?
Gallwitz 2012	1	490	0	487	2.2%	2.99 [0.12, 73.52]	+	?	?	?	+
Gerstein 2019	9	4949	7	4952	30.0%	1.29 [0.48, 3.46]	+	+	?	+	+
Holman 2017	5	7356	2	7396	8.6%	2.51 [0.49, 12.97]	+	+	?	+	+
Husain 2019	0	1591	2	1592	10.7%	0.20 [0.01, 4.17]	+	+	?	+	+
Kadowaki 2022	1	401	0	101	3.4%	0.76 [0.03, 18.80]	+	+	?	+	+
Lincoff 2023	2	8803	2	8801	8.6%	1.00 [0.14, 7.10]	+	+	?	+	?
Marso 2016	1	4668	2	4672	8.6%	0.50 [0.05, 5.52]	+	+	?	+	+
Marso 2016 a	6	1648	3	1649	12.9%	2.00 [0.50, 8.03]	+	+	?	+	+
Pfeffer 2015	0	3034	1	3034	6.5%	0.33 [0.01, 8.18]	+	+	?	+	+
Pi-Sunyer 2015	1	2481	0	1242	2.9%	1.50 [0.06, 36.91]	+	+	?	?	?
Pratley 2011	1	439	0	219	2.9%	1.50 [0.06, 37.01]	+	+	?	+	+
**Total (95% CI)**		**36448**		**34441**	**100.0%**	**1.25 [0.72, 2.16]**					
Total events	28		19								

Heterogeneity: Chi² = 4.22, df = 11 (P = 0.96); I² = 0%
Test for overall effect: Z = 0.80 (P = 0.42)

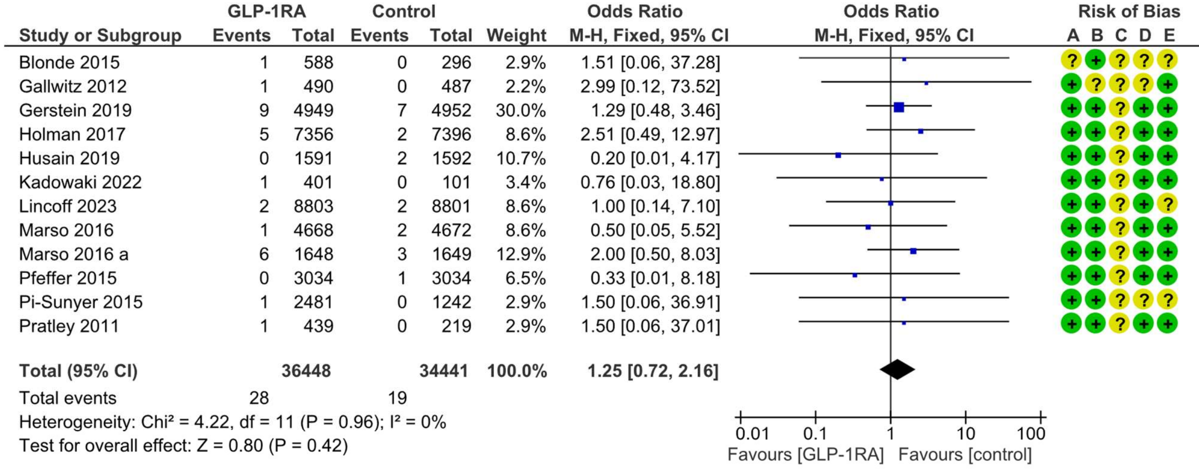

Risk of bias legend
(**A**) Randomization Process
(**B**) Assignment to Intervention
(**C**) Missing Outcome Data
(**D**) Measurement of the Outcome
(**E**) Bias in reported results

Fig. S16: Difference in risk for Gallbladder cancer and Cholangiocarcinoma between GLP1- RA and control

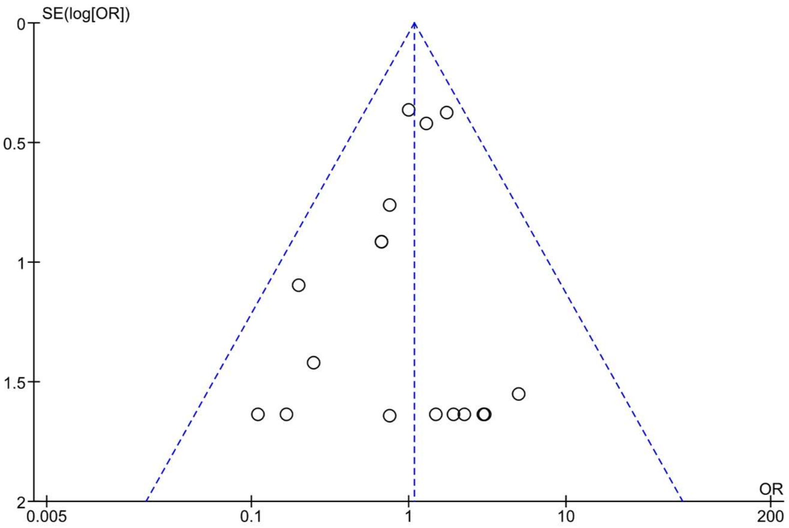

Fig. S17: funnel plot pancreas

Study or Subgroup	GLP-1RA Events	GLP-1RA Total	Control Events	Control Total	Weight	Odds Ratio M-H, Fixed, 95% CI	Risk of Bias A	B	C	D	E
Garvey 2020	1	195	0	197	0.8%	3.05 [0.12, 75.24]	+	?	+	?	?
Gerstein 2019	19	4949	11	4952	18.0%	1.73 [0.82, 3.64]	+	+	?	+	+
Gough 2015	1	414	0	413	0.8%	3.00 [0.12, 73.86]	+	?	?	+	+
Holman 2017	15	7356	15	7396	24.6%	1.01 [0.49, 2.06]	+	+	?	+	+
Husain 2019	3	1591	4	1592	6.6%	0.75 [0.17, 3.36]	+	+	?	+	+
Kaku 2018	1	480	1	121	2.6%	0.25 [0.02, 4.03]	+	+	?	?	?
Lincoff 2023	2	8803	3	8801	4.9%	0.67 [0.11, 3.99]	+	+	?	+	?
Marso 2016	2	4668	3	4672	4.9%	0.67 [0.11, 3.99]	+	+	?	+	+
Marso 2016 a	13	1648	10	1649	16.3%	1.30 [0.57, 2.98]	+	+	?	+	+
Miyagawa 2015	1	281	0	70	1.3%	0.75 [0.03, 18.71]	+	+	?	+	+
Nauck 2013	1	483	0	363	0.9%	2.26 [0.09, 55.64]	+	+	?	+	+
Pfeffer 2015	1	3034	5	3034	8.2%	0.20 [0.02, 1.71]	+	+	?	+	+
Pi-Sunyer 2015	0	2481	1	1242	3.3%	0.17 [0.01, 4.10]	+	+	?	?	?
Pratley 2011	1	439	0	219	1.1%	1.50 [0.06, 37.01]	+	+	?	+	+
Pratley 2019	1	284	0	182	1.0%	1.93 [0.08, 47.66]	+	+	?	+	+
Rosenstock 2019	0	1397	1	467	3.7%	0.11 [0.00, 2.74]	+	+	?	+	+
Ruff 2021	2	2074	0	2070	0.8%	5.00 [0.24, 104.11]	+	+	?	+	+
**Total (95% CI)**		**40577**		**37440**	**100.0%**	**1.08 [0.76, 1.53]**					
Total events	64		54								

Heterogeneity: Chi² = 11.44, df = 16 (P = 0.78); I² = 0%

Test for overall effect: Z = 0.42 (P = 0.68)

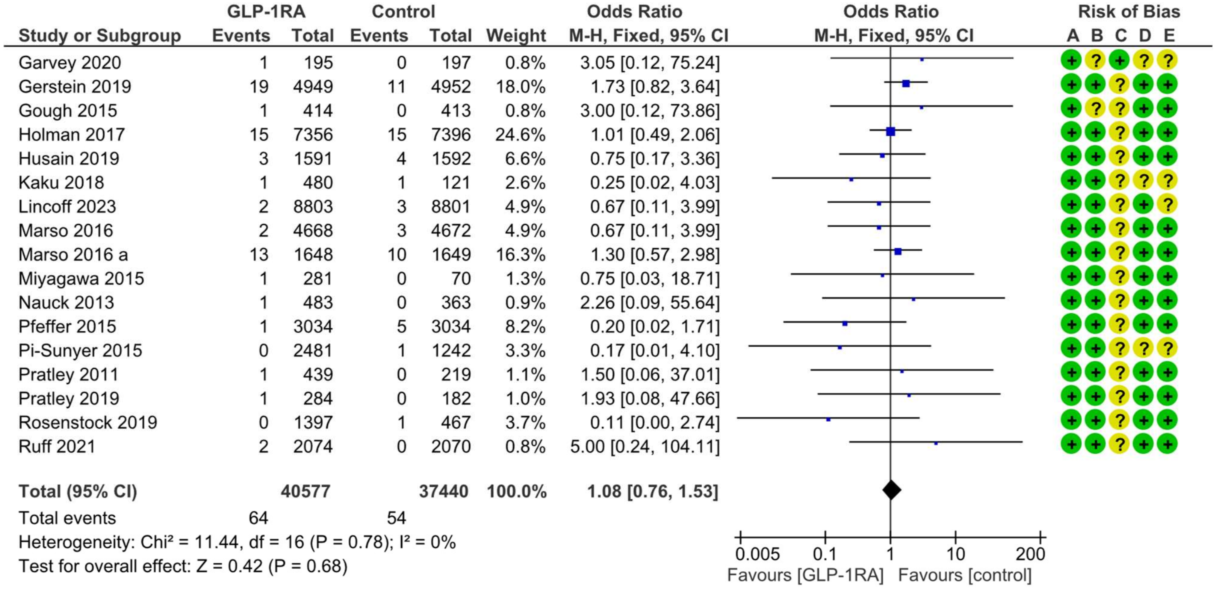

Risk of bias legend

(**A**) Randomization Process

(**B**) Assignment to Intervention

(**C**) Missing Outcome Data

(**D**) Measurement of the Outcome

(**E**) Bias in reported results

Fig. S18: Difference in risk for pancreas cancer between GLP1- RA and control

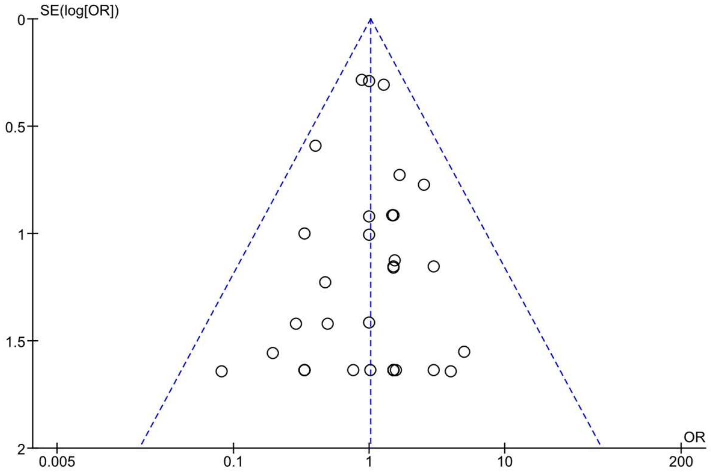

Fig. S19: Breast cancer , funnel plot

Study or Subgroup	GLP-1RA Events	GLP-1RA Total	Control Events	Control Total	Weight	Odds Ratio M-H, Fixed, 95% CI	A	B	C	D	E
Aroda 2019	0	506	1	504	1.2%	0.33 [0.01, 8.15]	+	?	?	+	+
Astrup 2013	1	369	0	193	0.5%	1.58 [0.06, 38.85]	+	?	?	?	+
Buse 2020	4	253	1	98	1.2%	1.56 [0.17, 14.12]	+	?	?	?	?
Davies 2021	1	805	0	402	0.5%	1.50 [0.06, 36.93]	+	+	?	?	?
Gallwitz 2012	3	490	2	487	1.6%	1.49 [0.25, 8.98]	+	?	?	?	+
Garber 2011	3	498	1	248	1.1%	1.50 [0.15, 14.47]	+	+	?	?	+
Garvey 2020	0	195	1	197	1.2%	0.34 [0.01, 8.27]	+	?	+	?	?
Garvey 2022	0	152	2	152	2.0%	0.20 [0.01, 4.15]	+	+	?	+	+
Gerstein 2019	23	4949	26	4952	21.2%	0.88 [0.50, 1.55]	+	+	?	+	+
Giorgino 2015	1	275	2	262	1.7%	0.47 [0.04, 5.26]	+	?	?	+	+
Gough 2015	1	414	0	413	0.4%	3.00 [0.12, 73.86]	+	?	?	+	+
Holman 2017	24	7356	19	7396	15.5%	1.27 [0.70, 2.32]	+	+	?	+	+
Husain 2019	0	1591	1	1592	1.2%	0.33 [0.01, 8.19]	+	+	?	+	+
Kaku 2018	1	480	0	121	0.7%	0.76 [0.03, 18.78]	+	+	?	?	?
Knop 2023	2	334	2	333	1.6%	1.00 [0.14, 7.12]	+	+	?	+	+
Lincoff 2023	4	8803	10	8801	8.2%	0.40 [0.13, 1.27]	+	+	?	+	?
Marso 2016	5	4668	3	4672	2.5%	1.67 [0.40, 6.99]	+	+	?	+	+
Marso 2016 a	24	1648	24	1649	19.4%	1.00 [0.57, 1.77]	+	+	?	+	+
Miyagawa 2015	0	281	1	70	2.0%	0.08 [0.00, 2.04]	+	+	?	+	+
O' Neill 2018	1	103	0	136	0.3%	4.00 [0.16, 99.08]	+	+	?	+	?
Pfeffer 2015	3	3034	2	3034	1.6%	1.50 [0.25, 8.99]	+	+	?	+	+
Pi-Sunyer 2015	10	2481	2	1242	2.2%	2.51 [0.55, 11.47]	+	+	?	?	?
Pratley 2011	1	439	0	219	0.5%	1.50 [0.06, 37.01]	+	+	?	+	+
Pratley 2019	1	284	0	142	0.5%	1.51 [0.06, 37.25]	+	+	?	+	+
Rodbard 2019	1	410	1	409	0.8%	1.00 [0.06, 16.00]	+	+	?	+	+
Rosenstock 2019	2	1397	2	467	2.5%	0.33 [0.05, 2.37]	+	+	?	+	+
Rubino 2022	3	253	2	170	1.9%	1.01 [0.17, 6.10]	+	+	?	+	+
Unger 2022	3	980	1	984	0.8%	3.02 [0.31, 29.07]	+	?	?	?	?
Wadden 2013	2	212	0	210	0.4%	5.00 [0.24, 104.77]	+	+	?	+	+
Wadden 2021	1	407	1	204	1.1%	0.50 [0.03, 8.03]	+	+	?	+	+
Wang 2019	1	515	0	259	0.5%	1.51 [0.06, 37.27]	+	?	?	?	+
Weinstock 2015	1	606	1	177	1.3%	0.29 [0.02, 4.67]	+	?	?	+	+
Wilding 2021	3	1306	1	655	1.1%	1.51 [0.16, 14.50]	+	+	?	+	+
Zinman 2019	1	547	0	184	0.6%	1.01 [0.04, 24.97]	+	+	?	+	+
**Total (95% CI)**		**47041**		**41034**	**100.0%**	**1.02 [0.79, 1.31]**					
Total events	131		109								

Heterogeneity: Chi² = 16.73, df = 33 (P = 0.99); I² = 0%

Test for overall effect: Z = 0.13 (P = 0.90)

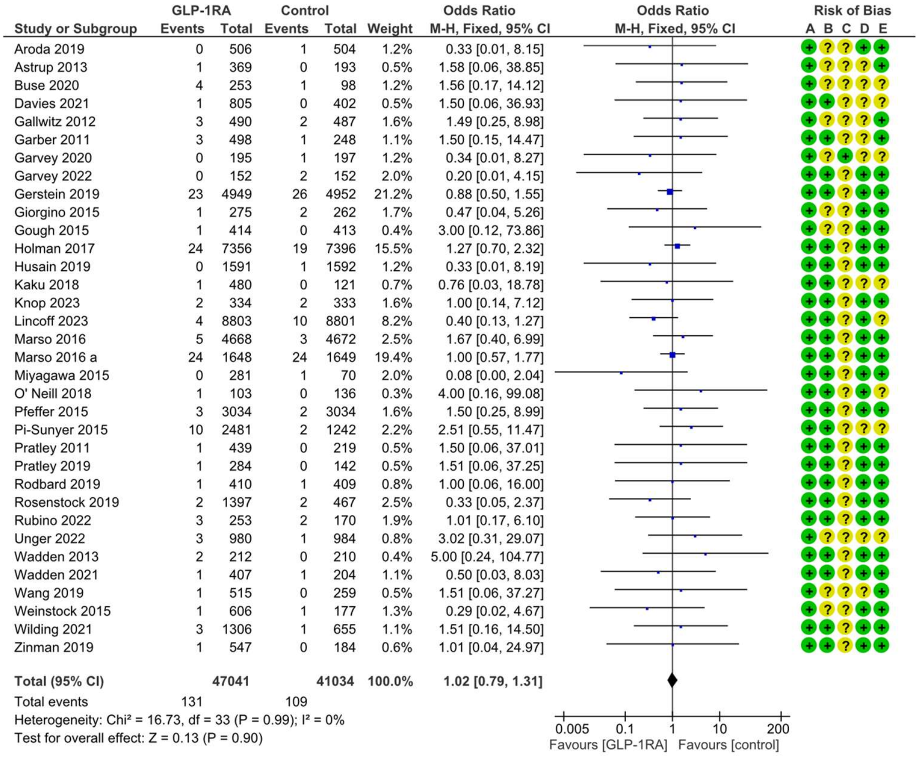

Risk of bias legend

(**A**) Randomization Process

(**B**) Assignment to Intervention

(**C**) Missing Outcome Data

(**D**) Measurement of the Outcome

(**E**) Bias in reported results

Fig. S20: Difference in risk for Breast cancer between GLP1- RA and control

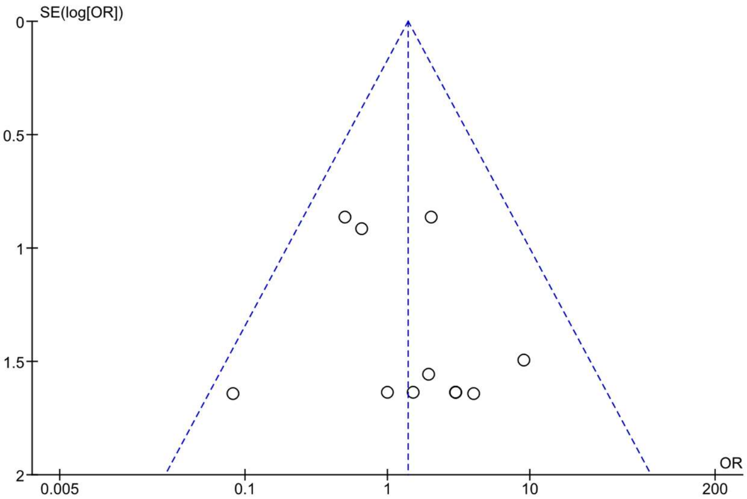

Fig. S21: Ovary cancer , funnel plot

Study or Subgroup	GLP-1RA Events	GLP-1RA Total	Control Events	Control Total	Weight	Odds Ratio M-H, Fixed, 95% CI	Risk of Bias A	B	C	D	E
Aroda 2019	1	506	0	506	3.1%	3.01 [0.12, 73.96]	+	?	?	+	+
Buse 2020	2	253	0	98	4.5%	1.96 [0.09, 41.15]	+	?	?	?	?
Gerstein 2019	2	4949	4	4952	25.1%	0.50 [0.09, 2.73]	+	+	?	+	+
Holman 2017	4	7356	2	7396	12.5%	2.01 [0.37, 10.98]	+	+	?	+	+
Marso 2016	1	4668	0	4672	3.1%	3.00 [0.12, 73.74]	+	+	?	+	+
Marso 2016 a	2	1648	3	1649	18.8%	0.67 [0.11, 3.99]	+	+	?	+	+
Miyagawa 2015	0	281	1	70	15.0%	0.08 [0.00, 2.04]	+	+	?	+	+
O' Neill 2018	1	103	0	136	2.7%	4.00 [0.16, 99.08]	+	+	?	+	?
Pfeffer 2015	4	3034	0	3034	3.1%	9.01 [0.48, 167.46]	+	+	?	+	+
Pratley 2019	1	284	0	142	4.2%	1.51 [0.06, 37.25]	+	+	?	+	+
Rosenstock 2019	1	1397	0	467	4.7%	1.00 [0.04, 24.70]	+	+	?	+	+
Wadden 2013	1	212	0	210	3.1%	2.99 [0.12, 73.71]	+	+	?	+	+
**Total (95% CI)**		**24691**		**23332**	**100.0%**	**1.38 [0.72, 2.65]**					
Total events	20		10								

Heterogeneity: Chi² = 7.93, df = 11 (P = 0.72); I^2 = 0%
Test for overall effect: Z = 0.98 (P = 0.33)

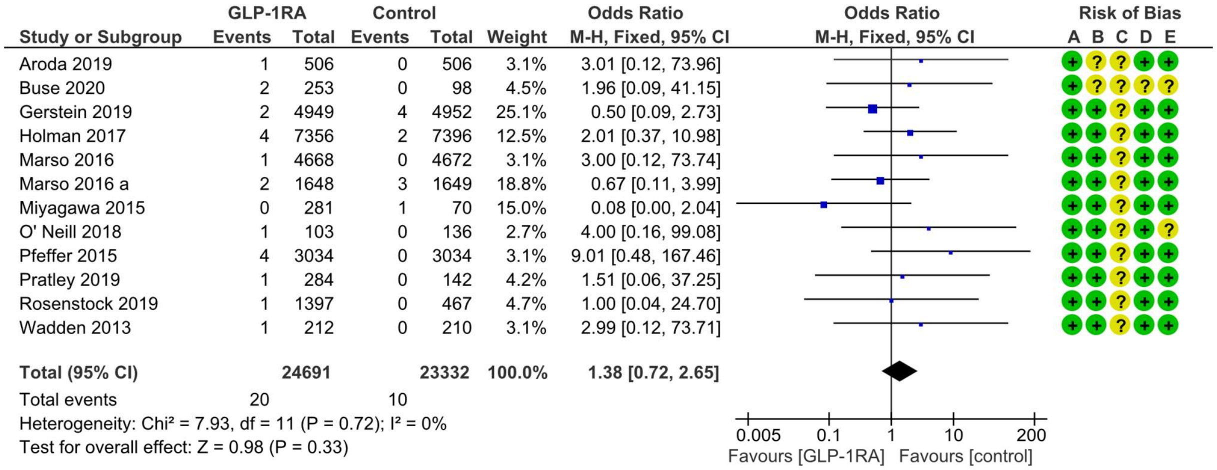

Risk of bias legend
(**A**) Randomization Process
(**B**) Assignment to Intervention
(**C**) Missing Outcome Data
(**D**) Measurement of the Outcome
(**E**) Bias in reported results

Fig. S22: Difference in risk for ovary cancer between GLP1- RA and control

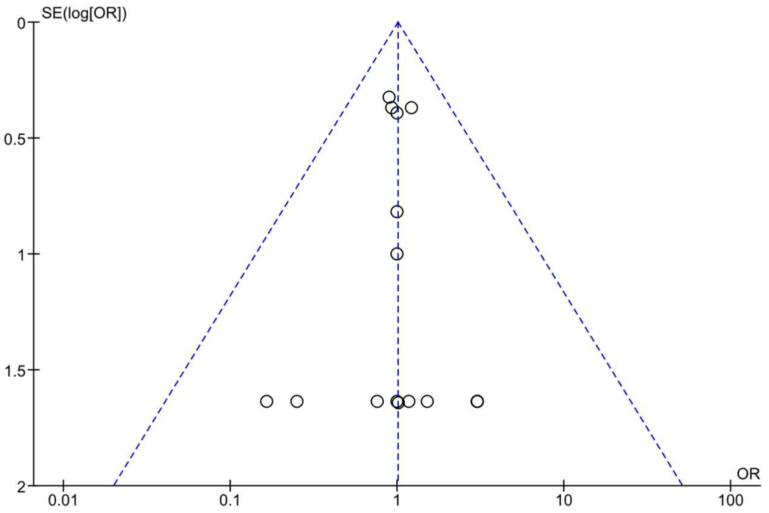

Figure S23: funnel plot for incidence of kidney cancer

Study or Subgroup	GLP-1RA Events	GLP-1RA Total	Control Events	Control Total	Weight	Odds Ratio M-H, Fixed, 95% CI	Risk of Bias A	B	C	D	E
Buse 2020	1	253	0	98	1.0%	1.17 [0.05, 28.97]	+	?	?	?	?
Garvey 2020	1	195	0	197	0.7%	3.05 [0.12, 75.24]	+	?	+	?	?
Gerstein 2019	18	4949	20	4952	26.7%	0.90 [0.48, 1.70]	+	+	?	+	+
Holman 2017	14	7356	15	7396	20.0%	0.94 [0.45, 1.95]	+	+	?	+	+
Husain 2019	1	1591	0	1592	0.7%	3.00 [0.12, 73.79]	+	+	?	+	+
Kadowaki 2022	1	401	0	101	1.1%	0.76 [0.03, 18.80]	+	+	?	+	+
Lincoff 2023	16	8803	13	8801	17.4%	1.23 [0.59, 2.56]	+	+	?	+	?
Marso 2016	2	4668	2	4672	2.7%	1.00 [0.14, 7.11]	+	+	?	+	+
Marso 2016 a	13	1648	13	1649	17.3%	1.00 [0.46, 2.17]	+	+	?	+	+
Nauck 2013	0	483	1	363	2.3%	0.25 [0.01, 6.15]	+	+	?	+	+
Pfeffer 2015	3	3034	3	3034	4.0%	1.00 [0.20, 4.96]	+	+	?	+	+
Pratley 2011	0	439	1	219	2.7%	0.17 [0.01, 4.08]	+	+	?	+	+
Rodbard 2019	1	410	0	409	0.7%	3.00 [0.12, 73.86]	+	+	?	+	+
Rosenstock 2019	1	1397	0	467	1.0%	1.00 [0.04, 24.70]	+	+	?	+	+
Rubino 2022	1	253	0	85	1.0%	1.02 [0.04, 25.17]	+	+	?	+	+
Wang 2019	1	515	0	259	0.9%	1.51 [0.06, 37.27]	+	?	?	?	+
**Total (95% CI)**		**36395**		**34294**	**100.0%**	**1.01 [0.73, 1.39]**					
Total events	74		68								

Heterogeneity: Chi² = 3.84, df = 15 (P = 1.00); I² = 0%
Test for overall effect: Z = 0.04 (P = 0.97)

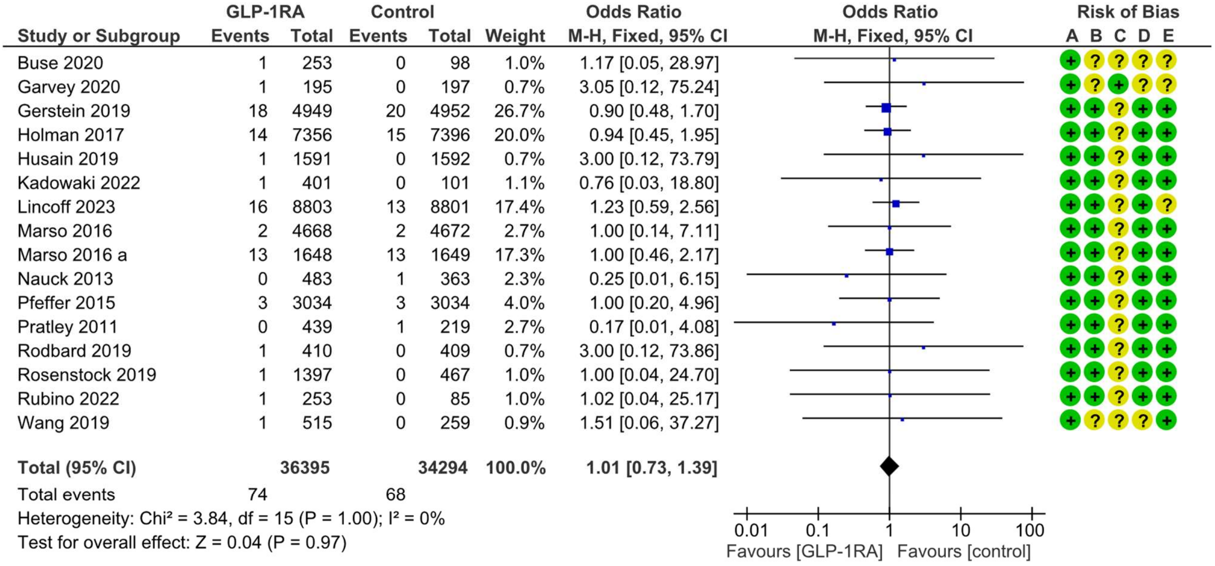

Risk of bias legend
(**A**) Randomization Process
(**B**) Assignment to Intervention
(**C**) Missing Outcome Data
(**D**) Measurement of the Outcome
(**E**) Bias in reported results

Figure S24: Difference in risk for *kidney cancer* between GLP1- RA and control

Study or Subgroup	GLP-1RA Events	GLP-1RA Total	Control Events	Control Total	Weight	Odds Ratio M-H, Fixed, 95% CI	Risk of Bias A	B	C	D	E
Gerstein 2019	2	4949	2	4952	23.5%	1.00 [0.14, 7.11]	+	+	?	+	+
Holman 2017	1	7356	1	7396	11.7%	1.01 [0.06, 16.08]	+	+	?	+	+
Lincoff 2023	3	8803	2	8801	23.6%	1.50 [0.25, 8.98]	+	+	?	+	?
Marso 2016 a	2	1648	3	1649	35.3%	0.67 [0.11, 3.99]	+	+	?	+	+
Pfeffer 2015	1	3034	0	3034	5.9%	3.00 [0.12, 73.70]	+	+	?	+	+
**Total (95% CI)**		**25790**		**25832**	**100.0%**	**1.12 [0.44, 2.82]**					
Total events	9		8								

Heterogeneity: Chi² = 0.81, df = 4 (P = 0.94); I² = 0%
Test for overall effect: Z = 0.24 (P = 0.81)

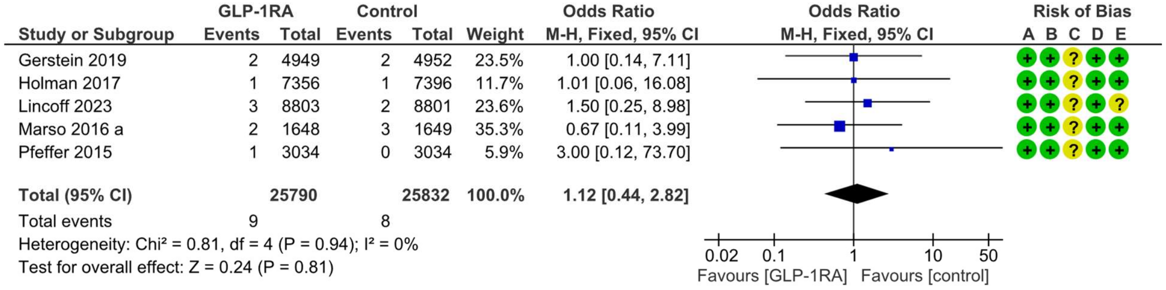

Risk of bias legend
(**A**) Randomization Process
(**B**) Assignment to Intervention
(**C**) Missing Outcome Data
(**D**) Measurement of the Outcome
(**E**) Bias in reported results

Figure S25: Difference in risk for Meningioma between GLP1- RA and control

Study or Subgroup	GLP-1RA Events	GLP-1RA Total	Control Events	Control Total	Weight	Odds Ratio M-H, Fixed, 95% CI	Risk of Bias A	B	C	D	E
Gerstein 2019	6	4949	3	4952	14.1%	2.00 [0.50, 8.01]	+	+	?	+	+
Giorgino 2015	1	275	0	262	2.4%	2.87 [0.12, 70.74]	+	?	?	+	+
Holman 2017	5	7356	5	7396	23.5%	1.01 [0.29, 3.47]	+	+	?	+	+
Husain 2019	1	1591	0	1592	2.4%	3.00 [0.12, 73.79]	+	+	?	+	+
Lincoff 2023	5	8803	6	8801	28.3%	0.83 [0.25, 2.73]	+	+	?	+	?
Marso 2016 a	3	1648	5	1649	23.5%	0.60 [0.14, 2.51]	+	+	?	+	+
Pfeffer 2015	1	3034	0	3034	2.4%	3.00 [0.12, 73.70]	+	+	?	+	+
Rosenstock 2019	1	1397	0	467	3.5%	1.00 [0.04, 24.70]	+	+	?	+	+
**Total (95% CI)**		**29053**		**28153**	**100.0%**	**1.14 [0.64, 2.04]**					
Total events	23		19								

Heterogeneity: Chi² = 2.74, df = 7 (P = 0.91); I² = 0%
Test for overall effect: Z = 0.44 (P = 0.66)

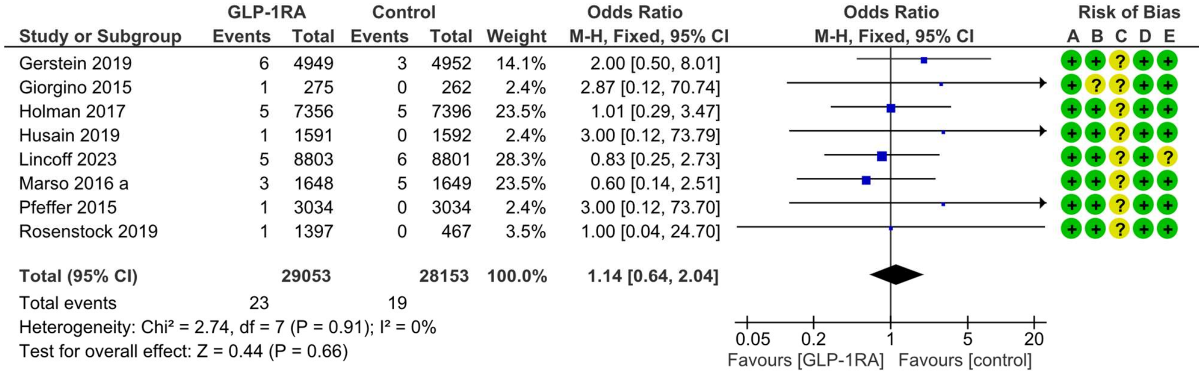

Risk of bias legend
(**A**) Randomization Process
(**B**) Assignment to Intervention
(**C**) Missing Outcome Data
(**D**) Measurement of the Outcome
(**E**) Bias in reported results

Figure S26: Difference in risk for Multiple Myeloma between GLP1- RA and control

Study or Subgroup	GLP-1RA Events	GLP-1RA Total	Control Events	Control Total	Weight	Odds Ratio M-H, Fixed, 95% CI
Aroda 2019	1	506	0	506	0.2%	3.01 [0.12, 73.96]
Blonde 2015	1	588	0	296	0.3%	1.51 [0.06, 37.28]
Buse 2020	2	253	0	98	0.3%	1.96 [0.09, 41.15]
Davies 2021	0	805	2	402	1.5%	0.10 [0.00, 2.08]
Gallwitz 2012	2	490	1	487	0.4%	1.99 [0.18, 22.04]
Garber 2011	1	498	0	248	0.3%	1.50 [0.06, 36.92]
Gerstein 2019	39	4949	42	4952	18.4%	0.93 [0.60, 1.44]
Giorgino 2015	1	275	0	262	0.2%	2.87 [0.12, 70.74]
Gough 2015	1	414	1	413	0.4%	1.00 [0.06, 16.00]
Holman 2017	21	7356	27	7396	11.9%	0.78 [0.44, 1.38]
Husain 2019	3	1591	4	1592	1.8%	0.75 [0.17, 3.36]
Knop 2023	1	334	0	333	0.2%	3.00 [0.12, 73.91]
Lincoff 2023	71	8803	70	8801	30.7%	1.01 [0.73, 1.41]
Marso 2016	6	4668	7	4672	3.1%	0.86 [0.29, 2.55]
Marso 2016 a	26	1648	44	1649	19.2%	0.58 [0.36, 0.95]
Miyagawa 2015	0	281	1	70	1.1%	0.08 [0.00, 2.04]
Nahra 2022	1	110	0	112	0.2%	3.08 [0.12, 76.48]
Nauck 2007	0	253	1	248	0.7%	0.33 [0.01, 8.03]
Nauck 2013	1	483	0	363	0.3%	2.26 [0.09, 55.64]
Perkovic 2024	9	1767	6	1766	2.6%	1.50 [0.53, 4.23]
Pfeffer 2015	12	3034	8	3034	3.5%	1.50 [0.61, 3.68]
Pi-Sunyer 2015	1	2481	0	1242	0.3%	1.50 [0.06, 36.91]
Rosenstock 2019	2	1397	0	467	0.3%	1.68 [0.08, 34.95]
Unger 2022	1	980	1	984	0.4%	1.00 [0.06, 16.08]
Weinstock 2015	0	606	1	177	1.0%	0.10 [0.00, 2.39]
Wilding 2021	1	1306	0	655	0.3%	1.51 [0.06, 37.03]
Zinman 2019	1	362	0	184	0.3%	1.53 [0.06, 37.77]
**Total (95% CI)**		**46238**		**41409**	**100.0%**	**0.91 [0.75, 1.10]**
Total events	205		216			

Heterogeneity: Chi² = 16.18, df = 26 (P = 0.93); I² = 0%

Test for overall effect: Z = 0.99 (P = 0.32)

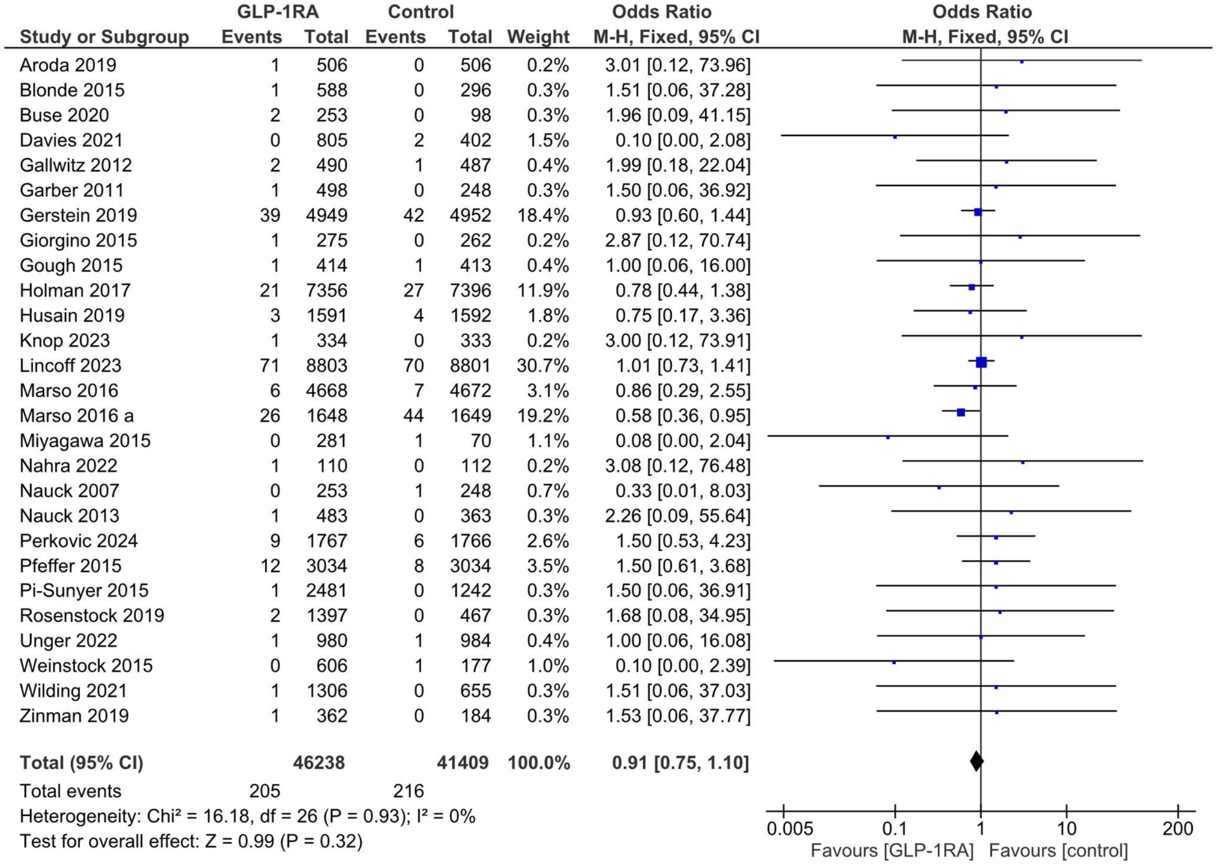

Figure S27: Difference in risk for Prostate cancer between GLP1- RA and comparators

Study or Subgroup	GLP-1RA Events	GLP-1RA Total	Control Events	Control Total	Weight	Odds Ratio M-H, Fixed, 95% CI
Davies 2021	0	805	1	402	4.6%	0.17 [0.01, 4.09]
Garber 2011	1	498	0	248	1.5%	1.50 [0.06, 36.92]
Gerstein 2019	5	4949	8	4952	18.4%	0.63 [0.20, 1.91]
Giorgino 2015	1	275	0	262	1.2%	2.87 [0.12, 70.74]
Holman 2017	6	7356	13	7396	29.8%	0.46 [0.18, 1.22]
Husain 2019	0	1591	2	1592	5.7%	0.20 [0.01, 4.17]
Lincoff 2023	7	8803	5	8801	11.5%	1.40 [0.44, 4.41]
Marso 2016	2	4668	1	4672	2.3%	2.00 [0.18, 22.09]
Marso 2016 a	8	1648	5	1649	11.4%	1.60 [0.52, 4.91]
Pfeffer 2015	1	3034	1	3034	2.3%	1.00 [0.06, 15.99]
Pi-Sunyer 2015	1	2481	0	1242	1.5%	1.50 [0.06, 36.91]
Pratley 2011	1	439	0	219	1.5%	1.50 [0.06, 37.01]
Rosenstock 2019	1	1397	0	467	1.7%	1.00 [0.04, 24.70]
Umpierrez 2014	1	269	0	268	1.1%	3.00 [0.12, 73.97]
Weinstock 2015	0	606	1	177	5.3%	0.10 [0.00, 2.39]
**Total (95% CI)**		**38819**		**35381**	**100.0%**	**0.84 [0.55, 1.31]**
Total events	35		37			

Heterogeneity: Chi² = 9.42, df = 14 (P = 0.80); I² = 0%

Test for overall effect: Z = 0.75 (P = 0.45)

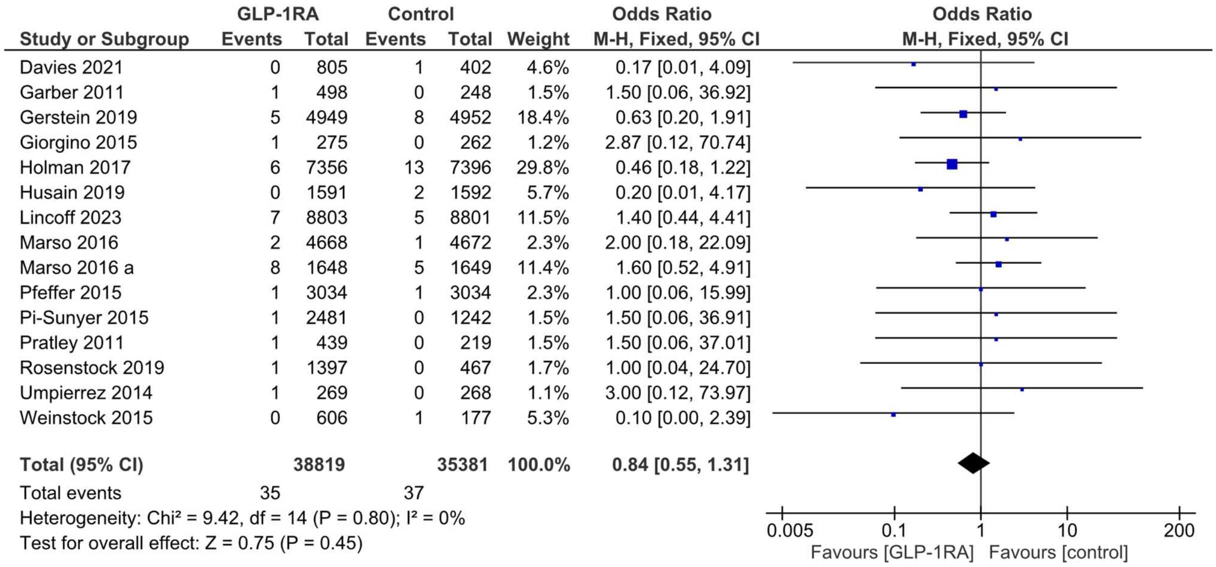

Figure S28: Difference in risk for ORL and Salivary Cancer between GLP1- RA and comparators

Study or Subgroup	GLP-1RA Events	GLP-1RA Total	Control Events	Control Total	Weight	Odds Ratio M-H, Fixed, 95% CI
Davies 2021	1	805	0	402	1.1%	1.50 [0.06, 36.93]
Gerstein 2019	14	4949	7	4952	11.8%	2.00 [0.81, 4.97]
Holman 2017	15	7356	12	7396	20.2%	1.26 [0.59, 2.69]
Husain 2019	2	1591	2	1592	3.4%	1.00 [0.14, 7.11]
Kaku 2018	10	480	1	121	2.6%	2.55 [0.32, 20.14]
Lincoff 2023	13	8803	7	8801	11.8%	1.86 [0.74, 4.66]
Marso 2016	3	4668	5	4672	8.4%	0.60 [0.14, 2.51]
Marso 2016 a	10	1648	13	1649	21.8%	0.77 [0.34, 1.76]
Nauck 2013	1	483	0	363	1.0%	2.26 [0.09, 55.64]
Pfeffer 2015	3	3034	6	3034	10.1%	0.50 [0.12, 2.00]
Pi-Sunyer 2015	2	2481	1	1242	2.2%	1.00 [0.09, 11.05]
Rosenstock 2019	1	1397	0	467	1.3%	1.00 [0.04, 24.70]
Umpierrez 2014	1	269	0	268	0.8%	3.00 [0.12, 73.97]
Wilding 2021	0	1306	1	655	3.4%	0.17 [0.01, 4.10]
**Total (95% CI)**		**39270**		**35614**	**100.0%**	**1.18 [0.84, 1.68]**
Total events	76		55			

Heterogeneity: Chi^2 = 8.17, df = 13 (P = 0.83); I^2 = 0%
Test for overall effect: Z = 0.96 (P = 0.34)

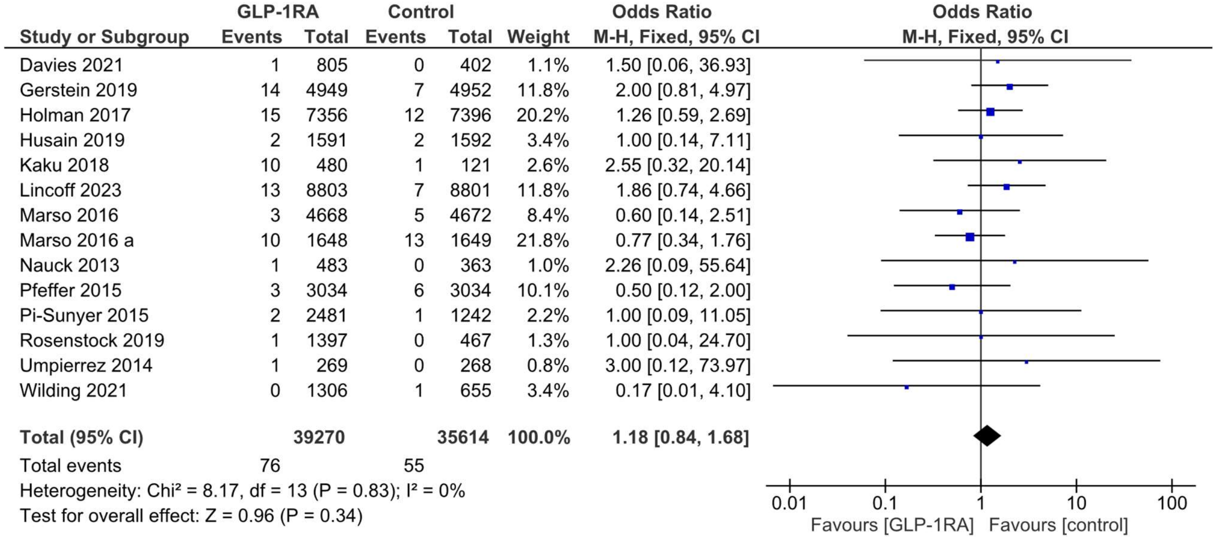

Figure S29: Difference in risk for Linfoma -LLC between GLP1- RA and comparators

Study or Subgroup	GLP-1RA Events	GLP-1RA Total	Control Events	Control Total	Weight	Odds Ratio M-H, Fixed, 95% CI
Davies 2021	1	805	0	402	5.1%	1.50 [0.06, 36.93]
Gerstein 2019	0	4949	1	4952	11.4%	0.33 [0.01, 8.19]
Holman 2017	1	7356	4	7396	30.4%	0.25 [0.03, 2.25]
Lincoff 2023	3	8803	2	8801	15.2%	1.50 [0.25, 8.98]
Marso 2016	0	4668	1	4672	11.4%	0.33 [0.01, 8.19]
Marso 2016 a	2	1648	2	1649	15.2%	1.00 [0.14, 7.11]
Nahra 2022	0	110	1	112	11.3%	0.34 [0.01, 8.35]
**Total (95% CI)**		**28339**		**27984**	**100.0%**	**0.65 [0.27, 1.53]**
Total events	7		11			

Heterogeneity: Chi^2 = 2.51, df = 6 (P = 0.87); I^2 = 0%
Test for overall effect: Z = 0.99 (P = 0.32)

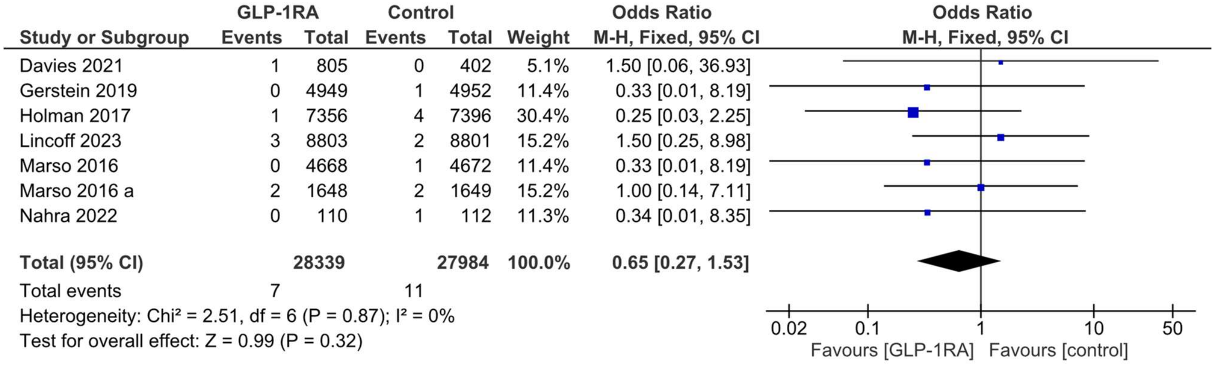

Figure S30: Difference in risk for LMC between GLP1- RA and comparators

Study or Subgroup	GLP-1RA Events	GLP-1RA Total	Control Events	Control Total	Weight	Odds Ratio M-H, Random, 95% CI
Gerstein 2019	1	4949	0	4952	14.5%	3.00 [0.12, 73.72]
Holman 2017	1	7356	1	7396	19.4%	1.01 [0.06, 16.08]
Husain 2019	0	1591	1	1592	14.5%	0.33 [0.01, 8.19]
Lincoff 2023	0	8803	2	8801	16.1%	0.20 [0.01, 4.16]
Marso 2016 a	1	1648	1	1649	19.3%	1.00 [0.06, 16.01]
Pi-Sunyer 2015	2	2481	0	1242	16.1%	2.51 [0.12, 52.23]
**Total (95% CI)**		**26828**		**25632**	**100.0%**	**0.90 [0.26, 3.03]**
Total events	5		5			

Heterogeneity: Tau^2 = 0.00; Chi^2 = 2.31, df = 5 (P = 0.80); I^2 = 0%
Test for overall effect: Z = 0.18 (P = 0.86)

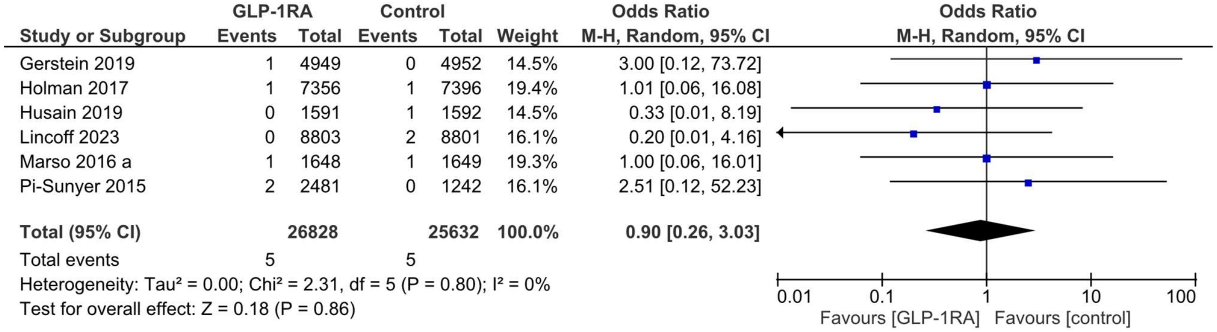

Figure S31: Difference in risk for LMA between GLP1- RA and comparators

Study or Subgroup	GLP-1RA Events	GLP-1RA Total	Control Events	Control Total	Weight	Odds Ratio M-H, Random, 95% CI	Risk of Bias A	B	C	D	E
Gerstein 2019	3	4949	0	4952	9.2%	7.01 [0.36, 135.72]	+	+	?	+	+
Holman 2017	3	7356	4	7396	35.9%	0.75 [0.17, 3.37]	+	+	?	+	+
Husain 2019	1	1591	1	1592	10.5%	1.00 [0.06, 16.01]	+	+	?	+	+
Lincoff 2023	2	8803	3	8801	25.2%	0.67 [0.11, 3.99]	+	+	?	+	?
Marso 2016 a	1	1648	1	1649	10.5%	1.00 [0.06, 16.01]	+	+	?	+	+
Pi-Sunyer 2015	2	2481	0	1242	8.7%	2.51 [0.12, 52.23]	+	+	?	?	?
**Total (95% CI)**		**26828**		**25632**	**100.0%**	**1.06 [0.43, 2.59]**					
Total events	12		9								

Heterogeneity: Tau^2 = 0.00; Chi^2 = 2.41, df = 5 (P = 0.79); I^2 = 0%
Test for overall effect: Z = 0.12 (P = 0.90)

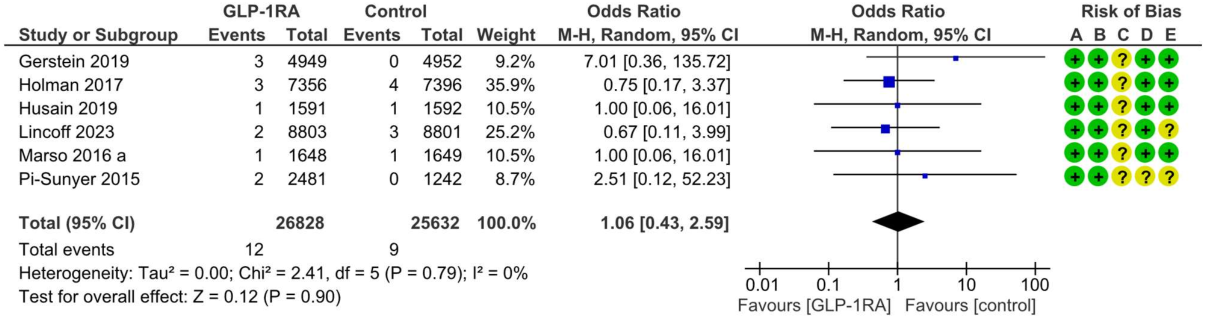

Figure S32: Difference in risk for Mieolproliferative Syndroms between GLP1- RA and comparators

Study or Subgroup	GLP-1RA Events	GLP-1RA Total	Control Events	Control Total	Weight	Odds Ratio M-H, Fixed, 95% CI	Risk of Bias A	B	C	D	E
Aroda 2019	0	506	1	506	2.9%	0.33 [0.01, 8.19]	+	?	?	+	+
Gerstein 2019	3	4949	5	4952	9.8%	0.60 [0.14, 2.51]	+	+	?	+	+
Holman 2017	17	7356	13	7396	25.3%	1.32 [0.64, 2.71]	+	+	?	+	+
Husain 2019	0	1591	1	1592	2.9%	0.33 [0.01, 8.19]	+	+	?	+	+
Knop 2023	1	334	1	333	2.0%	1.00 [0.06, 16.01]	+	+	?	+	+
Lincoff 2023	18	8803	17	8801	33.3%	1.06 [0.55, 2.06]	+	+	?	+	?
Marso 2016	1	4668	0	4672	1.0%	3.00 [0.12, 73.74]	+	+	?	+	+
Marso 2016 a	5	1648	10	1649	19.5%	0.50 [0.17, 1.46]	+	+	?	+	+
Pfeffer 2015	1	3034	1	3034	2.0%	1.00 [0.06, 15.99]	+	+	?	+	+
Pi-Sunyer 2015	1	2481	0	1242	1.3%	1.50 [0.06, 36.91]	+	+	?	?	?
**Total (95% CI)**		**35370**		**34177**	**100.0%**	**0.95 [0.64, 1.41]**					
Total events	47		49								

Heterogeneity: Chi² = 4.06, df = 9 (P = 0.91); I² = 0%
Test for overall effect: Z = 0.26 (P = 0.80)

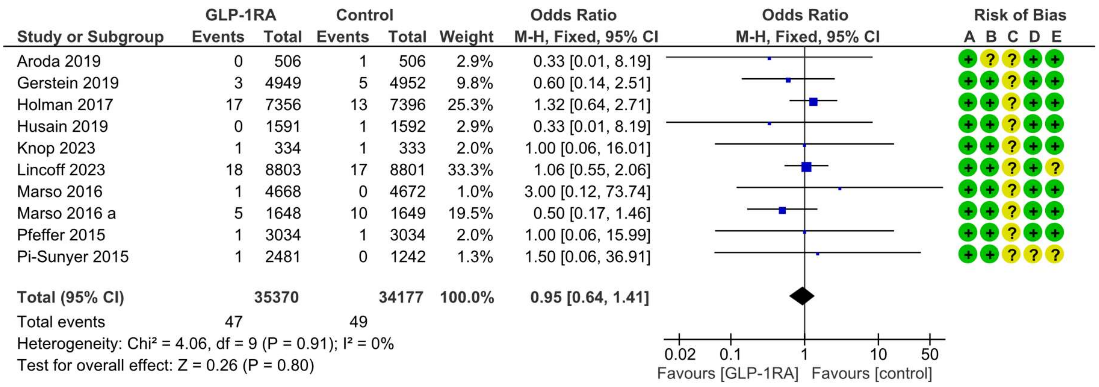

Figure S33: Difference in risk for Squamous Skin cancer between GLP1- RA and comparators

Study or Subgroup	GLP-1RA Events	GLP-1RA Total	Control Events	Control Total	Weight	Odds Ratio M-H, Fixed, 95% CI
Buse 2020	3	253	0	98	2.3%	2.75 [0.14, 53.78]
Garvey 2020	0	195	1	197	4.8%	0.34 [0.01, 8.27]
Gerstein 2019	5	4949	6	4952	19.5%	0.83 [0.25, 2.73]
Gough 2015	1	414	0	413	1.6%	3.00 [0.12, 73.86]
Holman 2017	11	7356	9	7396	29.1%	1.23 [0.51, 2.97]
Husain 2019	1	1591	1	1592	3.2%	1.00 [0.06, 16.01]
Kosiborod 2023	1	310	0	306	1.6%	2.97 [0.12, 73.21]
Lincoff 2023	1	8803	2	8801	6.5%	0.50 [0.05, 5.51]
Marso 2016	2	4668	0	4672	1.6%	5.01 [0.24, 104.31]
Marso 2016 a	12	1648	8	1649	25.8%	1.50 [0.61, 3.69]
Pfeffer 2015	2	3034	0	3034	1.6%	5.00 [0.24, 104.26]
Pi-Sunyer 2015	1	2481	0	1242	2.2%	1.50 [0.06, 36.91]
**Total (95% CI)**		**35702**		**34352**	**100.0%**	**1.35 [0.84, 2.15]**
Total events	40		27			

Heterogeneity: Chi² = 4.28, df = 11 (P = 0.96); I² = 0%
Test for overall effect: Z = 1.25 (P = 0.21)

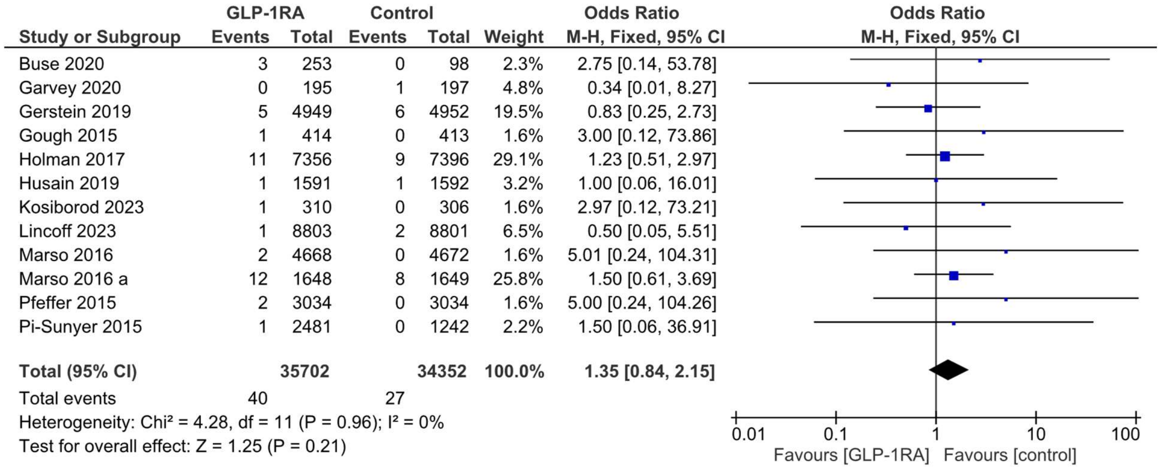

Figure S34: Difference in risk for Melanoma between GLP1- RA and comparators

Study or Subgroup	GLP-1RA Events	GLP-1RA Total	Control Events	Control Total	Weight	Odds Ratio M-H, Fixed, 95% CI
Aroda 2019	0	506	2	506	2.3%	0.20 [0.01, 4.16]
Blonde 2015	1	588	0	296	0.6%	1.51 [0.06, 37.28]
Buse 2020	0	253	1	98	2.0%	0.13 [0.01, 3.17]
Gallwitz 2012	1	490	1	487	0.9%	0.99 [0.06, 15.93]
Gerstein 2019	7	4949	11	4952	10.3%	0.64 [0.25, 1.64]
Holman 2017	42	7356	34	7396	31.6%	1.24 [0.79, 1.96]
Husain 2019	0	1591	1	1592	1.4%	0.33 [0.01, 8.19]
Knop 2023	0	334	2	333	2.3%	0.20 [0.01, 4.14]
Kosiborod 2023	9	310	1	306	0.9%	9.12 [1.15, 72.42]
Lincoff 2023	21	8803	36	8801	33.7%	0.58 [0.34, 1.00]
Marso 2016 a	14	1648	7	1649	6.5%	2.01 [0.81, 4.99]
Pfeffer 2015	0	3034	5	3034	5.2%	0.09 [0.01, 1.64]
Rodbard 2019	0	410	1	409	1.4%	0.33 [0.01, 8.17]
Rubino 2022	1	253	0	85	0.7%	1.02 [0.04, 25.17]
**Total (95% CI)**		**30525**		**29944**	**100.0%**	**0.92 [0.70, 1.21]**
Total events	96		102			

Heterogeneity: Chi² = 19.32, df = 13 (P = 0.11); I² = 33%
Test for overall effect: Z = 0.59 (P = 0.56)

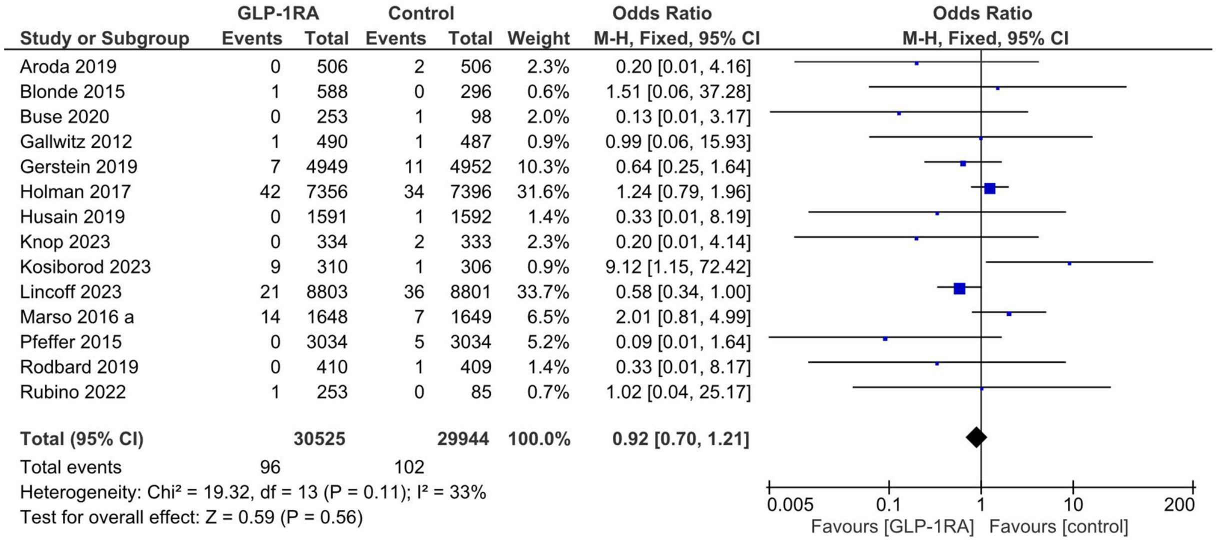

Figure S35: Difference in risk for Basal Skin cancer between GLP1- RA and comparators

Study or Subgroup	GLP-1RA Events	GLP-1RA Total	Control Events	Control Total	Weight	Odds Ratio M-H, Fixed, 95% CI	Risk of Bias A	B	C	D	E
Ahren 2017	1	818	0	407	0.6%	1.50 [0.06, 36.79]	+	+	?	+	+
Gallwitz 2012	0	490	3	487	3.2%	0.14 [0.01, 2.74]	+	?	?	?	+
Gerstein 2019	20	4949	23	4952	20.7%	0.87 [0.48, 1.59]	+	+	?	+	+
Giorgino 2015	1	275	0	262	0.5%	2.87 [0.12, 70.74]	+	?	?	+	+
Gough 2015	1	414	0	413	0.5%	3.00 [0.12, 73.86]	+	?	?	+	+
Holman 2017	23	7356	20	7396	18.0%	1.16 [0.63, 2.11]	+	+	?	+	+
Husain 2019	0	1591	2	1592	2.3%	0.20 [0.01, 4.17]	+	+	?	+	+
Kaku 2018	1	480	0	121	0.7%	0.76 [0.03, 18.78]	+	+	?	?	?
Lincoff 2023	32	8803	32	8801	28.9%	1.00 [0.61, 1.63]	+	+	?	+	?
Marso 2016	4	4668	3	4672	2.7%	1.33 [0.30, 5.97]	+	+	?	+	+
Marso 2016 a	20	1648	18	1649	16.1%	1.11 [0.59, 2.11]	+	+	?	+	+
O' Neill 2018	0	103	1	136	1.2%	0.44 [0.02, 10.82]	+	+	?	+	?
Pfeffer 2015	0	3034	3	3034	3.2%	0.14 [0.01, 2.76]	+	+	?	+	+
Tuttle 2018	1	383	0	194	0.6%	1.53 [0.06, 37.62]	+	?	?	?	+
Unger 2022	1	980	0	984	0.5%	3.02 [0.12, 74.11]	+	?	?	?	?
Wang 2019	1	515	0	259	0.6%	1.51 [0.06, 37.27]	+	?	?	?	+
**Total (95% CI)**		**36507**		**35359**	**100.0%**	**0.98 [0.75, 1.28]**					
Total events	106		105								

Heterogeneity: Chi² = 6.93, df = 15 (P = 0.96); I² = 0%
Test for overall effect: Z = 0.12 (P = 0.90)

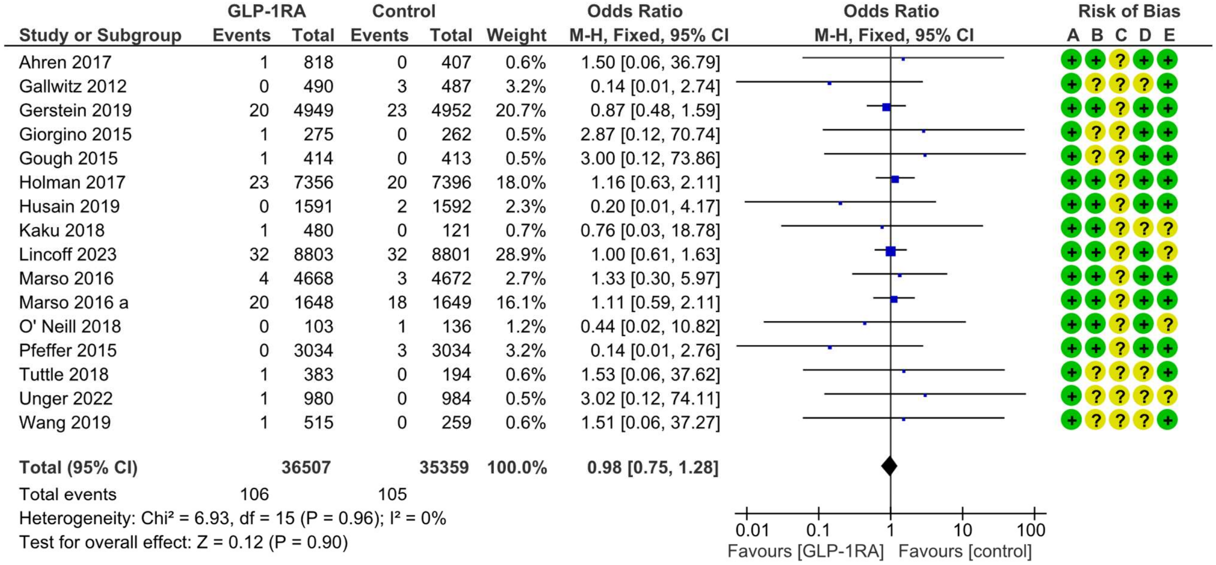

Risk of bias legend
(**A**) Randomization Process
(**B**) Assignment to Intervention
(**C**) Missing Outcome Data
(**D**) Measurement of the Outcome
(**E**) Bias in reported results

Figure S36: Difference in risk for Urothelial Bladder Cancer between GLP1- RA and comparators

Study or Subgroup	GLP-1RA Events	GLP-1RA Total	Control Events	Control Total	Weight	Odds Ratio M-H, Fixed, 95% CI
Aroda 2019	1	506	1	506	0.8%	1.00 [0.06, 16.03]
Blonde 2015	0	588	1	296	1.6%	0.17 [0.01, 4.12]
Buse 2020	1	253	0	98	0.6%	1.17 [0.05, 28.97]
Gallwitz 2012	1	490	0	487	0.4%	2.99 [0.12, 73.52]
Garvey 2022	0	152	1	152	1.2%	0.33 [0.01, 8.19]
Gerstein 2019	24	4949	21	4952	17.1%	1.14 [0.64, 2.06]
Holman 2017	33	7356	36	7396	29.2%	0.92 [0.57, 1.48]
Husain 2019	4	1591	1	1592	0.8%	4.01 [0.45, 35.92]
Kadowaki 2022	1	401	0	101	0.6%	0.76 [0.03, 18.80]
Kaku 2018	1	480	0	121	0.6%	0.76 [0.03, 18.78]
Lincoff 2023	6	8803	7	8801	5.7%	0.86 [0.29, 2.55]
Marso 2016	3	4668	5	4672	4.1%	0.60 [0.14, 2.51]
Marso 2016 a	26	1648	33	1649	26.5%	0.78 [0.47, 1.32]
Miyagawa 2015	1	281	0	70	0.6%	0.75 [0.03, 18.71]
Nauck 2013	1	483	0	363	0.5%	2.26 [0.09, 55.64]
Pfeffer 2015	5	3034	6	3034	4.9%	0.83 [0.25, 2.73]
Pratley 2019	1	285	0	142	0.5%	1.50 [0.06, 37.12]
Rodbard 2019	1	410	0	409	0.4%	3.00 [0.12, 73.86]
Unger 2022	0	980	4	984	3.7%	0.11 [0.01, 2.07]
**Total (95% CI)**		**37358**		**35825**	**100.0%**	**0.90 [0.70, 1.17]**
Total events	110		116			

Heterogeneity: Chi² = 7.99, df = 18 (P = 0.98); I² = 0%
Test for overall effect: Z = 0.78 (P = 0.44)

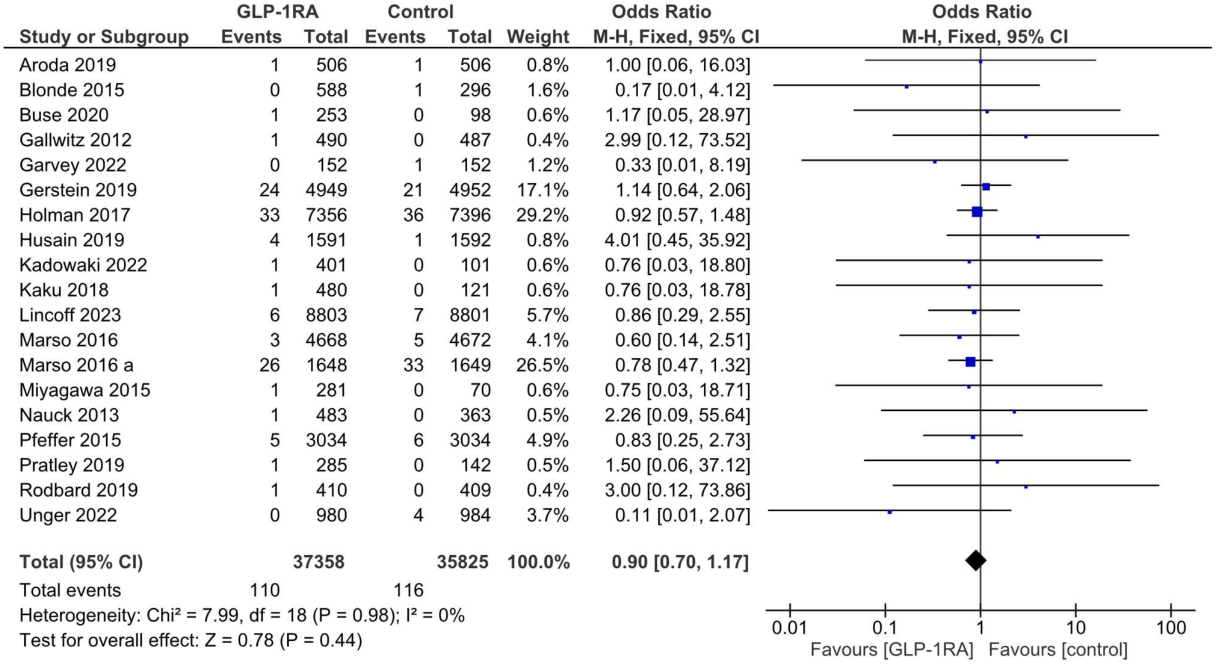

Figure S37: Non small cell pumonary between GLP1- RA and comparators

Study or Subgroup	GLP-1RA Events	GLP-1RA Total	Control Events	Control Total	Weight	Odds Ratio M-H, Fixed, 95% CI	Risk of Bias A	B	C	D	E
Gallwitz 2012	0	490	1	487	7.9%	0.33 [0.01, 8.14]	+	?	?	?	+
Garvey 2022	0	152	1	152	7.9%	0.33 [0.01, 8.19]	+	+	?	+	+
Gerstein 2019	2	4949	0	4952	2.6%	5.01 [0.24, 104.28]	+	+	?	+	+
Holman 2017	3	7356	2	7396	10.5%	1.51 [0.25, 9.03]	+	+	?	+	+
Husain 2019	0	1591	1	1592	7.9%	0.33 [0.01, 8.19]	+	+	?	+	+
Lincoff 2023	9	8803	6	8801	31.6%	1.50 [0.53, 4.22]	+	+	?	+	?
Marso 2016	2	4668	2	4672	10.5%	1.00 [0.14, 7.11]	+	+	?	+	+
Marso 2016 a	6	1648	3	1649	15.8%	2.00 [0.50, 8.03]	+	+	?	+	+
Pfeffer 2015	2	3034	1	3034	5.3%	2.00 [0.18, 22.08]	+	+	?	+	+
**Total (95% CI)**		**32691**		**32735**	**100.0%**	**1.37 [0.76, 2.48]**					
Total events	24		17								

Heterogeneity: Chi² = 3.48, df = 8 (P = 0.90); I² = 0%
Test for overall effect: Z = 1.04 (P = 0.30)

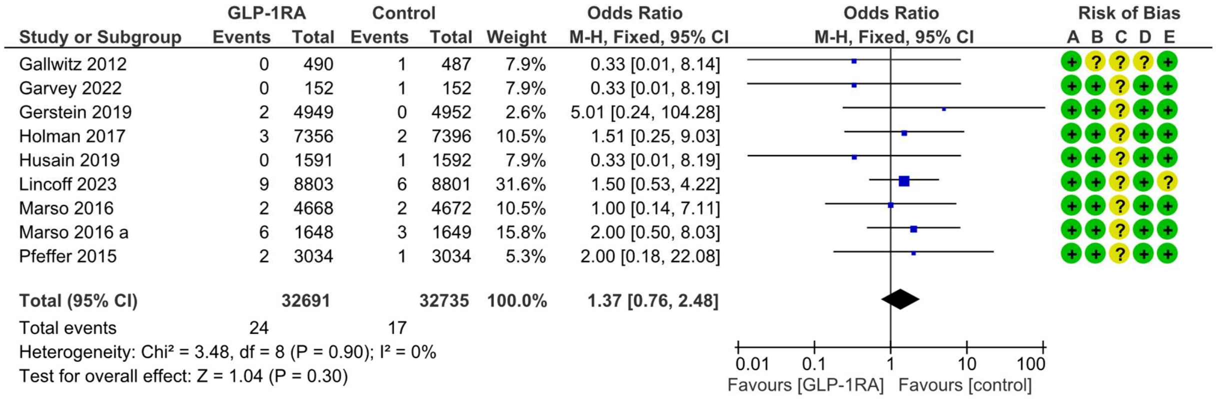

Risk of bias legend
(**A**) Randomization Process
(**B**) Assignment to Intervention
(**C**) Missing Outcome Data
(**D**) Measurement of the Outcome
(**E**) Bias in reported results

Figure S38: Difference in risk for Non small cell pumonary between GLP1- RA and comparators

Study or Subgroup	GLP-1RA Events	GLP-1RA Total	Control Events	Control Total	Weight	Odds Ratio M-H, Random, 95% CI
Ahren 2017	0	818	1	407	6.9%	0.17 [0.01, 4.07]
Davies 2021	1	805	0	402	6.9%	1.50 [0.06, 36.93]
Gerstein 2019	3	4949	5	4952	34.6%	0.60 [0.14, 2.51]
Holman 2017	0	7356	1	7396	6.9%	0.34 [0.01, 8.23]
Jaiswal 2015	1	22	0	24	6.7%	3.42 [0.13, 88.40]
Lincoff 2023	0	8803	1	8801	6.9%	0.33 [0.01, 8.18]
Marso 2016	3	4668	0	4672	8.1%	7.01 [0.36, 135.76]
Marso 2016 a	1	1648	1	1649	9.2%	1.00 [0.06, 16.01]
Pfeffer 2015	1	3034	0	3034	6.9%	3.00 [0.12, 73.70]
Rosenstock 2019	1	1397	0	467	6.9%	1.00 [0.04, 24.70]
**Total (95% CI)**		**33500**		**31804**	**100.0%**	**0.90 [0.39, 2.08]**
Total events	11		9			

Heterogeneity: Tau² = 0.00; Chi² = 5.30, df = 9 (P = 0.81); I² = 0%
Test for overall effect: Z = 0.25 (P = 0.80)

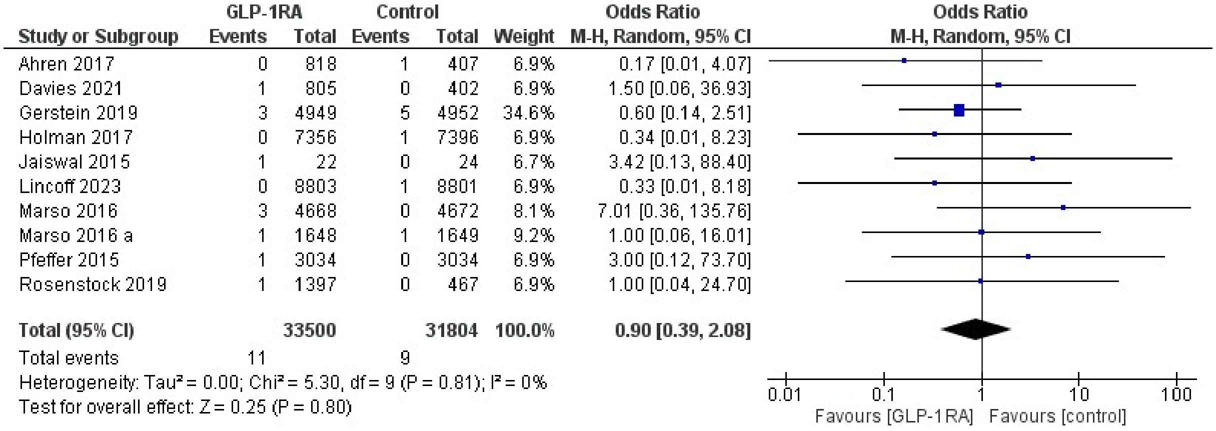

Figure S39: Difference in risk for Neuroendocrine Tumors between GLP1- RA and comparators

Study or Subgroup	GLP-1RA Events	GLP-1RA Total	Control Events	Control Total	Weight	Odds Ratio M-H, Fixed, 95% CI	Risk of Bias A	B	C	D	E
Gerstein 2019	0	4949	1	4952	13.6%	0.33 [0.01, 8.19]	+	+	?	+	+
Holman 2017	1	7356	0	7396	4.5%	3.02 [0.12, 74.06]	+	+	?	+	+
Lincoff 2023	4	8803	3	8801	27.3%	1.33 [0.30, 5.96]	+	+	?	+	?
Marso 2016	1	4668	4	4672	36.4%	0.25 [0.03, 2.24]	+	+	?	+	+
Marso 2016 a	3	1648	2	1649	18.2%	1.50 [0.25, 9.00]	+	+	?	+	+
**Total (95% CI)**		**27424**		**27470**	**100.0%**	**0.91 [0.39, 2.14]**					
Total events	9		10								

Heterogeneity: Chi² = 2.80, df = 4 (P = 0.59); I² = 0%
Test for overall effect: Z = 0.22 (P = 0.83)

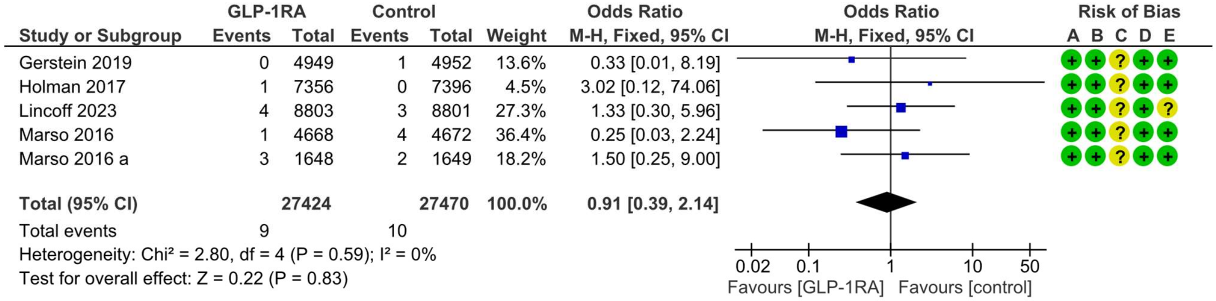

Risk of bias legend
(**A**) Randomization Process
(**B**) Assignment to Intervention
(**C**) Missing Outcome Data
(**D**) Measurement of the Outcome
(**E**) Bias in reported results

Figure S40: Difference in risk for Parathyroid Adenoma between GLP1- RA and comparators

Study or Subgroup	GLP-1RA Events	GLP-1RA Total	Control Events	Control Total	Weight	Odds Ratio M-H, Random, 95% CI	Risk of Bias A	B	C	D	E
Giorgino 2015	1	275	0	262	31.5%	2.87 [0.12, 70.74]	+	?	?	+	+
Lincoff 2023	3	8803	0	8801	36.9%	7.00 [0.36, 135.55]	+	+	?	+	?
Pfeffer 2015	0	3034	1	3034	31.6%	0.33 [0.01, 8.18]	+	+	?	+	+
**Total (95% CI)**		**12112**		**12097**	**100.0%**	**2.02 [0.33, 12.21]**					
Total events	4		1								

Heterogeneity: Tau² = 0.00; Chi² = 1.95, df = 2 (P = 0.38); I² = 0%
Test for overall effect: Z = 0.77 (P = 0.44)

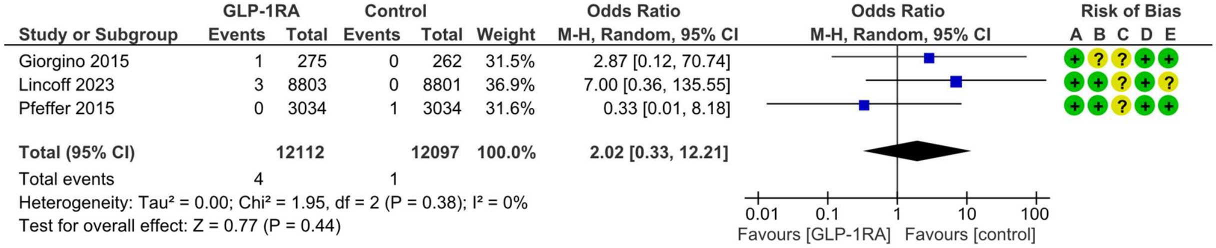

Risk of bias legend
(**A**) Randomization Process
(**B**) Assignment to Intervention
(**C**) Missing Outcome Data
(**D**) Measurement of the Outcome
(**E**) Bias in reported results

Figure S41: Difference in risk for Testis Cancer between GLP1- RA and comparators

Study or Subgroup	GLP-1RA Events	GLP-1RA Total	Control Events	Control Total	Weight	Odds Ratio M-H, Fixed, 95% CI	Risk of Bias A	B	C	D	E
Gerstein 2019	2	4949	3	4952	29.1%	0.67 [0.11, 3.99]	+	+	?	+	+
Holman 2017	0	7356	2	7396	24.2%	0.20 [0.01, 4.19]	+	+	?	+	+
Husain 2019	2	1591	0	1592	4.8%	5.01 [0.24, 104.43]	+	+	?	+	+
Marso 2016	1	4668	1	4672	9.7%	1.00 [0.06, 16.01]	+	+	?	+	+
Marso 2016 a	1	1648	0	1649	4.8%	3.00 [0.12, 73.79]	+	+	?	+	+
Pfeffer 2015	1	3034	0	3034	4.8%	3.00 [0.12, 73.70]	+	+	?	+	+
Weinstock 2015	0	606	1	177	22.5%	0.10 [0.00, 2.39]	+	?	?	+	+
**Total (95% CI)**		**23852**		**23472**	**100.0%**	**0.90 [0.37, 2.17]**					
Total events	7		7								

Heterogeneity: Chi² = 5.22, df = 6 (P = 0.52); I² = 0%
Test for overall effect: Z = 0.24 (P = 0.81)

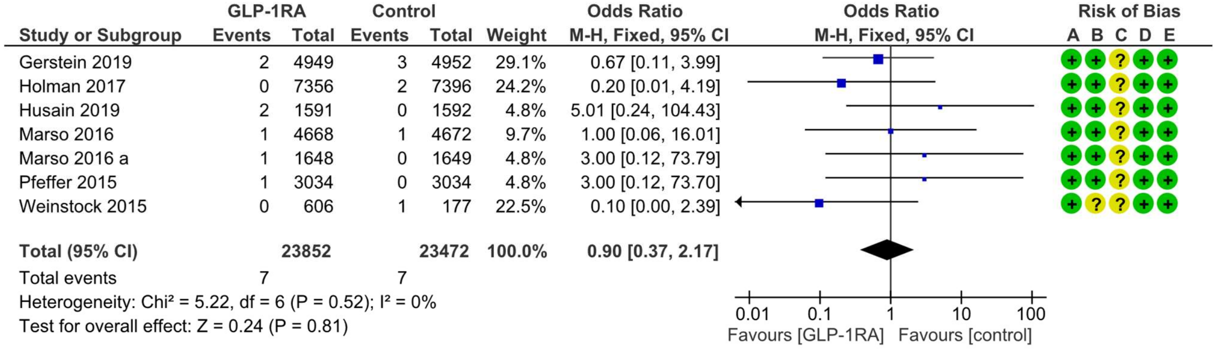

Risk of bias legend
(**A**) Randomization Process
(**B**) Assignment to Intervention
(**C**) Missing Outcome Data
(**D**) Measurement of the Outcome
(**E**) Bias in reported results

Figure S43: Difference in risk for Pituitary Adenoma between GLP1- RA and comparators
